# Potential Cosmetic Applications of the Combined Extract of *Panax ginseng*, *Ganoderma lucidum*, *Cordyceps militaris*, and Several Asian Plants

**DOI:** 10.1111/jocd.70343

**Published:** 2025-08-05

**Authors:** Wenlong Wang, Ming Zhong, Ronghua Liu, Shichun Zhao, Xianwen Wei, Qiwan Zheng, Qingyu Zhong, Yujie Shen, Feng He

**Affiliations:** ^1^ Lasur Cosmetics Co., Ltd. Huzhou China; ^2^ College of Pharmacy Jiangxi University of Chinese Medicine Nanchang China

**Keywords:** cell culture, computer modeling, cosmetic formulation, herbal synergy, multitargets efficacy, skin barrier

## Abstract

**Objective:**

Although bioactive compounds from single herbs are extensively explored in cosmetics, the synergistic potential of herbal combinations remains understudied. This study aimed to evaluate the stability of a combined extract of *Panax ginseng* , *Ganoderma lucidum*, *Cordyceps militaris*, and several Asian plants (PGC), and its multifunctional efficacy for acne‐related skin dysfunction.

**Methods:**

PGC was analyzed using high‐performance liquid chromatography (HPLC) for batch consistency and bioactive quantification. Network pharmacology and molecular docking were used to identify active components and targets and assess binding affinities, respectively. In vitro assays were conducted to evaluate antibacterial activity, skin barrier repair, keratinocyte migration, reactive oxygen species (ROS) reduction, anti‐inflammatory effects, and inhibition of lipid accumulation. The safety was tested via cytotoxicity assessments.

**Results:**

HPLC analysis validated batch consistency and identified key bioactive constituents in PGC, including phenolic acids, flavonoids, and alkaloids. Integrated network pharmacology and molecular docking revealed multitarget mechanisms through the regulation of the IL‐17/TNF/NF‐κB axis pathway modulation. PGC exhibited potent antibacterial efficacy against acne‐associated pathogens (*Cutibacterium acne*, MIC = 25 μg/mL), restored skin barrier integrity (filaggrin, +235%; loricrin, +261%), and sodium dodecyl sulfate (SDS)‐induced damage (85%). Concurrently, PGC accelerated keratinocyte migration (40%), reduced ROS (45%) and abnormal lipid droplet content (60%), and attenuated inflammatory responses (40% nitric oxide (NO) inhibition) while maintaining biosafety (no cytotoxicity ≤ 200 μg/mL).

**Conclusion:**

PGC exemplified the translational potential of herbal compatibility, offering a multitargets solution for acne management through integrating antibacterial, barrier‐repair, anti‐inflammatory actions, and several other effects. This study established a network pharmacology‐guided framework for developing evidence‐based multitargets cosmetics.

## 1 Introduction

Skin diseases represent a significant global health burden, with conditions such as acne, atopic dermatitis, and psoriasis profoundly affecting the quality of life of patients through aesthetic, psychological, and systemic consequences [1, 2, 3]. Acne, the most prevalent dermatological disorder, involves complex mechanisms including *Cutibacterium acnes*‐driven inflammation, ROS activation, abnormal sebum secretion, and compromised skin barrier function, often leading to secondary inflammatory responses [4, 5, 6]. Current treatments relying on antibiotics and glucocorticoids face limitations due to drug resistance and adverse effects [7, 8], highlighting the need for safer multi‐target therapies.

Natural ingredients are gaining traction in skincare for their perceived safety and multi‐functional potential. Phytochemicals such as phenols and flavonoids exert synergistic antibacterial, anti‐inflammatory, and antioxidant properties [9, 10], aligning with the “multicomponent, multitarget” paradigm of botanical therapeutics. This study focused on a combination of underutilized Asian botanicals, *Panax ginseng* , *Ganoderma lucidum*, and *Cordyceps militaris* (PGC), leveraging their traditional applications through modern analytical approaches.

**FIGURE 1:**
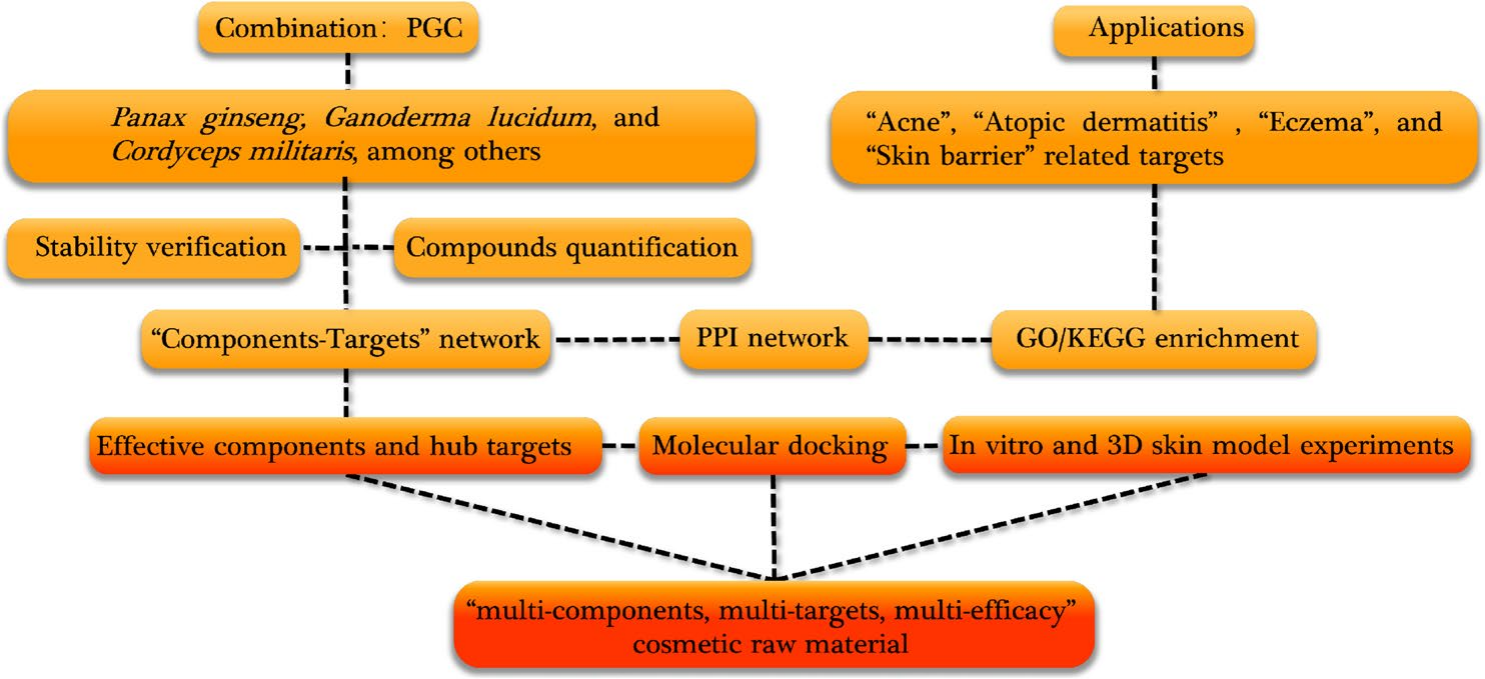
Research design.

Network pharmacology and molecular docking were integrated to systematically map the bioactive components of PGC against dermatologically relevant targets, establishing a “Components‐Targets‐Efficacy” framework. This strategy bridged the gap between traditional knowledge and evidence‐based cosmetic development. Subsequent validation through in vitro models confirmed the multifunctional efficacy of PGC against acne‐related skin dysfunction by achieving acne mitigation, anti‐inflammation, and barrier repair. Our approach provides a template for accelerating plant‐based cosmetic innovation through predictive analytics and mechanistic validation. The research design is shown below (Figure 1).

## 2 Materials and Methods

### 2.1 Acquisition of the Combined Extract

Dried raw materials, including *Panax ginseng* roots and rhizomes, dried fruiting bodies of *Ganoderma lucidum* and *Cordyceps militaris*, dried rhizomes of *Coptis chinensis*, dried fruits of *Forsythia suspensa* , dried flower buds of *Lonicera japonica* Thunb, and dried whole plants of *Taraxacum officinale* Wigg (Lasur Cosmetics, Zhejiang, China), were dehydrated (40°C, 48 h; residual moisture < 8%) and ground into 40‐mesh particles. A standardized mixture (1:1:1:1:1:1:1 w/w) was reflux‐extracted thrice with deionized water (1:10 w/v, 100°C° ± 2°C, 60 min per cycle). Combined filtrates were concentrated under reduced pressure to one‐fifth of the initial volume, and cold‐precipitated (4°C, 24 h). The supernatant was filtered through 5 μm cellulose acetate and 0.22 μm PVDF membranes, and lyophilized (−40°C prefreezing, 2 h; −20°C/300 μbar, 5 h; −10°C/300 μbar, 5 h; 10°C/300 μbar, 2 h; 25°C/300 μbar, 10 min; 42°C/300 μbar, 20 min; 38°C/300 μbar, 7 h) to yield the PGC powder (1.98% ± 0.06% w/w).

### 2.2 Combined Extract Stability Verification

A high‐performance liquid chromatography (HPLC) assay was performed using an Agilent 1220 HPLC instrument (Agilent Technologies, Santa Clara, CA, USA) and a quaternary pump (G4281B 1220 Infinity II GradPump, Agilent Technologies). The liquid chromatography parameters employed included the use of a ZORBAX Eclipse XDB‐C18 chromatographic column with dimensions of 250 mm × 4.6 mm and a particle size of 5 μm. The column was maintained at a temperature of 25°C, with an injection volume set at 5 μL. The mobile phase used in this study was a 0.2% solution of phosphoric acid (HPLC grade; Macklin Biochemical Technology, Shanghai, China), termed component A, in conjunction with acetonitrile (HPLC grade; Macklin Biochemical Technology, Shanghai, China), designated component B. The elution gradient is presented in Table 1, and detection was conducted at a wavelength of 254 nm.

**TABLE 1:** The elution gradient.

Time (min)	*A* (%)	*B* (%)	Flow (mL/min)
0	95	5	0.5
18	90	10	0.5
30	90	10	0.5
37	88	12	0.5
42	88	12	0.5
47	85	15	0.5
57	85	15	0.5
67	78	22	0.5
77	78	22	0.5
80	76	24	0.5
90	76	24	0.5
92	70	30	0.5
102	70	30	0.5

### 2.3 Quantification of Biologically Active Compounds

#### 2.3.1 Total Phenolic Acid Content

The total phenolic acid content (TPC) of the crude extract was evaluated utilizing a modified version of the methodology established by Yang et al. [11]. Initially, a 200 μL aliquot of the stock solution of reconstituted PGC lyophilized powder was diluted to a final volume of 10 mL with 0.1 mol/L HCl, followed by thorough agitation to create the test solution. Subsequently, 0.2 mL of this test solution was transferred into a 10‐mL volumetric flask, to which the following reagents were added in succession: 1.8 mL of absolute ethanol, 0.8 mL of a 0.3% SDS solution, and 0.40 mL of a mixed solution of 0.6% FeCl_3_ and 0.9% K_3_[Fe(CN)_6_] (in a volume ratio of 1:1). After thorough mixing, the solution was allowed to stand in the dark for 5 min, then diluted to 10 mL with 0.1 mol/L HCl, shaken well, and kept in the dark for an additional 20 min. Concurrently, a series of chlorogenic acid (≥ 98%, Standard Technology, Shanghai, China) standard solutions at varying concentrations were prepared to establish a calibration curve. The absorbance was measured at 724 nm using a UV spectrophotometer (Agilent Cary 60 UV–Vis, Agilent Technologies), and the concentration of the sample was calculated based on the calibration curve, in triplicate.

#### 2.3.2 Total Flavonoid Content

The total flavonoid content (TFC) of the crude extract was evaluated using a modified version of the methodology established by He et al. [12]. Initially, 100 μL of the stock solution of reconstituted PGC lyophilized powder was diluted to 10 mL with methanol, resulting in the test solution. A volume of 5 mL of this test solution was then transferred to a 10‐mL volumetric flask, to which 0.5 mL of a 5% sodium nitrite solution was added. The solution was thoroughly mixed and allowed to stand for 6 min. Following this, 0.5 mL of a 10% aluminum nitrate solution was added, mixed well, and allowed to stand for an additional 6 min. Subsequently, 3 mL of a 4% sodium hydroxide solution was added, and the solution was diluted to 10 mL with methanol, shaken vigorously, and allowed to stand in the dark for 10 min. Concurrently, a calibration curve was established using quercetin (≥ 98%, Desite Biotechnology, Chengdu, China) standard solutions of varying concentrations. The absorbance was measured at 510 nm, and the concentration of the sample was calculated based on the calibration curve, in triplicate.

#### 2.3.3 Total Alkaloid Content

The total alkaloid content (TAC) of the crude extract was evaluated using a modified version of the methodology established by Lan et al. [13]. Specifically, 100 μL of the stock solution of reconstituted PGC lyophilized powder was diluted to a final volume of 10 mL using a 0.275 mol/L hydrochloric acid solution, followed by thorough agitation to create the test solution. The absorbance of an appropriate aliquot of the test solution was measured at 430 nm. A 0.275 mol/L hydrochloric acid solution, prepared in an identical manner, served as a blank control and was subtracted from the readings to account for background interference. A calibration curve for berberine (≥ 98%, RENI Pharmaceutical Technology, Sichuan, China) was generated using standard solutions at varying concentrations under the same experimental conditions, facilitating the concentration (mg/mL) calculation of the test sample. Each sample was analyzed in triplicate to ensure the reliability of the results.

### 2.4 Network Pharmacology and Molecular Docking Analysis

#### 2.4.1 Prediction of Components and Targets

The components and associated targets of the combined extract were sourced from several databases, including the Swiss Target Prediction (http://www.swisstargetprediction.ch/), TCMSP (https://www.tcmsp‐e.com/), and DrugBank (https://go.drugbank.com/). The selection criteria for the components included bioavailability ≥ 30% and a drug‐likeness score ≥ 0.18 [14, 15, 16, 17].

Following the identification of related targets for each herb, redundant targets were removed to yield the therapeutic targets of the combined extract. These targets were systematically named according to their designations in the UniProt online protein database (https://www.uniprot.org/).

#### 2.4.2 Identification of Specific Targets

The key words “Acne,” “Atopic dermatitis,” “Eczema,” and “Skin barrier” were used to search for cosmetic applications‐related targets in Online Mendelian Inheritance in Man (OMIM, https://omim.org/) and GeneCards (https://www.genecards.org/). The targets obtained from the databases were combined, and the duplicated targets were deleted to obtain specific targets.

#### 2.4.3 Construction of Overlapping Targets

Component‐related and cosmetic‐related targets were combined to obtain an intersection of overlapping targets, indicating potential targets for cosmetic and skin care applications. The following networks and analyses were based on overlapping targets.

#### 2.4.4 Construction of a “PGC‐Components‐Targets‐Applications” Network

The components of the combined extract and their related targets were imported into Cytoscape 3.10.2 [18] to construct the network. The nodes of the network represented the combined extract, components, targets, or applications, whereas the edges between the nodes represented the interactions between them. The degree of a node was determined by the number of linked edges. The greater the degree value, the more important the node.

#### 2.4.5 Construction of a Protein–Protein Interaction Network

The overlapping component and cosmetic‐related targets were imported into the STRING database (https://string‐db.org/) to construct the Protein–Protein Interaction (PPI) network. In the STRING database, the organisms were limited to “ *Homo sapiens* ,” the minimum interaction threshold was “highest confidence > 0.9” and the disconnected nodes in the network were hidden as well. “Betweenness Centrality,” “Closeness Centrality,” “Degree,” “Eigenvector,” “LAC,” and “Network” were used as indicators to screen the more important targets with greater value than the median of these, and subsequently the PPI network was visualized using Cytoscape 3.10.2, in which nodes represented the combined extract and cosmetic‐related targets, whereas edges linked any two interactive target proteins.

#### 2.4.6 Enrichment Analysis of Gene Ontology Terms and Kyoto Encyclopedia of Genes and Genomes Pathway

The overlapping targets between the combined extract and specific cosmetic and skin care applications were imported into DAVID informatics (https://david.ncifcrf.gov/) for enrichment analysis of Gene Ontology (GO) terms (biological process, cellular component, and molecular function) and Kyoto Encyclopedia of Genes and Genomes (KEGG) pathways. GO terms are the most commonly used type of enrichment analysis in a gene set that helps researchers understand the common characteristics of genes in a gene set in terms of biological processes (BP), cellular components (CC), and molecular functions (MF). Pathway databases such as KEGG are commonly used resources for pathway enrichment analysis. This type of enrichment analysis focuses on the roles of genes in metabolic and signal transduction pathways. By detecting pathway enrichment in a gene set, researchers can better understand the functions and regulatory mechanisms of genes in an organism. The results of the enrichment analysis were visualized using SRPLOT (http://www.bioinformatics.com.cn/).

#### 2.4.7 Analysis of Interactions Between the Combined Extract and Its Targets Based on Molecular Docking

The interactions between the combined extract and hub targets were further validated using a semi‐flexible molecular docking approach. The components of the combined extract were subjected to energy minimization and three‐dimensional processing utilizing Chem3D 20.0, after which they were imported into AutoDockTools 1.5.7 for protonation and charge calculations, thereby preparing them as ligands. The protein molecules relevant to cosmetics, sourced from the Protein Data Bank (PDB), were pre‐processed using PyMOL, which involved the removal of water molecules, heteroatoms, unique ligands, and β‐chains. Subsequently, the same procedures applied to the ligands were repeated to prepare proteins as receptors. AutoDockTools encompass a comprehensive suite of methodologies for computational docking and virtual screening, facilitating structure‐based drug discovery and the investigation of fundamental mechanisms underlying biomolecular structure and function. The AutoGrid tool [19], included in AutoDockTools, enables visualization of a grid box on the protein for docking purposes. Following the adjustment of the grid parameters to establish an appropriate grid box, a semi‐flexible molecular docking operation was conducted to ascertain the potential binding positions and energies between the molecules and their respective targets.

### 2.5 Experimental Verifications

#### 2.5.1 Cells and Skin Model Culture

The HaCaT cell line was purchased from iCell Bioscience Co. Ltd. (Catalog No: iCell‐h066; Shanghai, China), the RAW 264.7 cell line was purchased from Wuhan Pricella Biotechnology Co. Ltd. (Catalog No: PC‐H2023012901; Wuhan, China), and the SZ95 cell line was sourced from Bluef Biotechnology Development Co. Ltd. (Catalog No: BFN60807569; Shanghai, China). All cell lines were maintained in tissue culture‐treated flasks (25 cm^2^) with high‐glucose Dulbecco's Modified Eagle Medium (Pricella Biotechnology, Wuhan, China), supplemented with 10% fetal bovine serum (FBS; Pricella Biotechnology) and 1% penicillin/streptomycin antibiotic. Adherent cells in culture were subjected to digestion using a 0.25% trypsin solution (Pricella Biotechnology) to generate a cell suspension. After acquisition of the 3D epidermal skin model (Biocell Biotechnology, Guangdong, China), the cells were placed in a 6‐well plate. A volume of 0.9 mL of a specialized culture medium, designated EpiRecovery (Biocell Biotechnology), was added to the cells and incubated for 1 h. Following this initial period, the medium was replaced with a fresh culture medium, and the skin model was cultured for an additional 18 h to ensure complete recovery. For ongoing culture of the skin model, an equivalent volume of another specialized culture medium, EpiGrowth (Biocell Biotechnology), was used. All cultures were incubated at 37°C in a humidified atmosphere containing 5% CO_2_.

PGC and water‐soluble compounds were solubilized in phosphate‐buffered saline (PBS; Pricella Biotechnology), whereas the remaining compounds were solubilized in dimethyl sulfoxide (DMSO; Solarbio Life Sciences, Beijing, China). Subsequently, all compounds were diluted in complete culture medium to achieve the desired test concentrations for biological assays. The final concentration of DMSO in all assays was consistently maintained ≤ 0.2%.

#### 2.5.2 Verification of Antibacterial Activity

The MICs of PGC against *Staphylococcus aureus* (ATCC25923, Lasur Cosmetics, Zhejiang, China), *Cutibacterium acnes* (ATCC6919, Lasur Cosmetics), and *Staphylococcus epidermidis* (ATCC14990, Lasur Cosmetics) were determined using a microdilution assay conducted in a 96‐well plate. Initially, a single colony was isolated from the preserved bacterial stock (HuanKai Microbial, Guangdong, China), inoculated onto an agar plate, and incubated at 37°C for 24 h. Subsequently, the isolated colony was transferred to Luria‐Bertani medium (HuanKai Microbial) until the culture reached the logarithmic growth phase, at which point the bacterial concentration was adjusted to 0.5 × 10^5^ CFU/mL. A volume of 95 μL of this bacterial suspension was added to each well of the 96‐well plate. Following this, 5 μL of various concentrations of PGC solution was added to each well, resulting in final concentrations of 200, 100, 50, 25, 12.5, and 6.25 μg/mL. Penicillin V potassium (Macklin Biochemical Technology, Shanghai, China) was used as the positive control. The 96‐well plate was subsequently incubated at 37°C for an additional 24 h, after which optical density (OD) was measured at 600 nm to assess the antibacterial efficacy of PGC.

#### 2.5.3 Determination of Cell Viability

Cell viability was assessed using the Cell Counting Kit‐8 (CCK‐8; Solarbio Life Sciences). After trypsinization, a cell suspension of 1 × 10^5^ cells/mL was inoculated into each well of a 96‐well plate and incubated for 24 h. The wells were categorized into eight distinct groups, each comprising six replicates, with the PGC group exhibiting six different concentration gradients. Following a 24 h incubation period, RAW 264.7 cells were treated with complete medium supplemented with 10% CCK‐8 for 90 min, whereas HaCaT and SZ95 cells were treated for 40 min. OD was measured using a microplate reader (YT‐1096A, Yetuo Technology, Shanghai, China) at a wavelength of 450 nm.

Cell viability was calculated using the formula:

(1) $$A=\left(B-C\right)/\left(D-C\right)\times 100\%$$

where *A* represents the cell viability in each well, *B* represents the absorbance value of each well, *C* represents the absorbance value of the blank control (without any cells), and *D* represents the absorbance value of the control group (untreated cells).

#### 2.5.4 Validation of Skin Structure Optimization

To assess the potential application of PGC in skincare, SDS (Macklin Biochemical Technology) was used to stimulate a 3D epidermal skin model [20]. Hematoxylin and eosin (HE) staining (Solarbio Life Sciences) and immunofluorescence techniques were employed in vitro to evaluate the barrier protective effects of PGC. Initially, the resuscitated model was transferred to a 6‐well plate and pretreated with 0.9 mL of EpiGrowth culture medium. Subsequently, 25 μL of SDS solution at a final concentration of 0.2% was applied to the surface of the model group, whereas 50 μM WY14643 (APE × BIO Technology, Shanghai, China) served as a positive control. According to the previous research [21, 22], the final PGC concentrations were set at 2, 1, and 0.5 mg/mL. The drug‐containing solution was uniformly distributed across the surface of the model and incubated at 37°C and 5% CO_2_ for 24 h.

HE staining was used to observe the pathological alterations in the 3D epidermal skin model slices following SDS stimulation, while changes in the levels of barrier‐related protein indicators, filaggrin (FLG) and loricrin (LOR), were assessed to evaluate the reparative efficacy and barrier protection of the test substance. After fixation with 4% paraformaldehyde, the skin models from different groups were placed in an embedding box and rinsed with running water overnight. Paraffin sections were prepared by dehydration, paraffin embedding, and slicing. The paraffin sections were dewaxed, hydrated, and stained with hematoxylin and eosin, followed by dehydration to achieve transparency, after which they were examined microscopically to compare changes among the groups. Following the same dewaxing and hydration procedures, the sections were subjected to antigen retrieval using 0.01 M sodium citrate solution (Aladdin Scientific, Shanghai, China) under high pressure. After blocking with peroxidase and serum, sections were incubated with appropriate primary and secondary antibodies (Thermo Fisher Scientific, Shanghai, China). Finally, Hoechst staining (Beyotime Biotechnology) was used to visualize the cell nuclei, and the fluorescence intensity of each group was quantified using an inverted fluorescence microscope.

#### 2.5.5 Validation of Cell Migration, Antioxidant, and Soothing Properties

Cell migration capacity was assessed using a cell scratch assay [23]. Cells were cultured to the appropriate density (6 × 10^5^ cells/mL) and subsequently inoculated into a 6‐well plate, with six auxiliary lines delineated at the bottom to facilitate positioning. Following a 24 h incubation period, the cells achieved 100% confluence, resulting in the formation of a cell monolayer. A 200‐μL pipette tip was employed to create a scratch in the cell layer within each well. Each well was washed once with 2 mL of 1× PBS to eliminate the displaced cells. The positive control group received 2 mL of complete medium supplemented with 10% FBS, whereas the negative control group was treated with 2 mL of complete medium containing 1% FBS. The experimental groups were administered 2 mL of complete medium containing different PGC concentrations along with 1% FBS. Images were captured immediately after the treatment. Subsequent imaging was performed 16 h after the scratch was made. To ensure consistency in the area of image collection, the circular field of the microscope eyepiece was positioned such that it was tangential to the auxiliary horizontal line located at the bottom of the well, and at least eight fields of vision were considered. The area of the cell scratch was quantified using the Image‐J software.

The migration rate was calculated using the formula:

(2) $$A=\left(B-C\right)/B\times 100\%$$

where *A* represents the migration rate in each well, *B* represents the scratch area in each well at 0 h, and *C* represents the scratch area in each well at 16 h.

An in vitro method for evaluating oxidative damage was formulated in accordance with established protocols [24]. HaCaT cells were cultured at a density of 1 × 10^5^ cells/mL in a 96‐well plate, with a pretreatment procedure conducted after 24 h of incubation. Vitamin C (Sinopharm, Beijing, China) at 60 μg/mL served as a positive control. Oxidative damage was induced by 3 mmol/L hydrogen peroxide (Sinopharm) 4 h before the assessment. ROS levels were measured using a dichlorofluorescein diacetate (DCFH‐DA, Beyotime Biotechnology) fluorescent probe to evaluate the degree of oxidative damage, and 4′,6‐Diamidino‐2‐phenylindole (DAPI; Solarbio Life Sciences) was used to visualize the cell nuclei. For each group, six images were captured under an inverted fluorescence microscope, and the fluorescence intensity of each field was subjected to semiquantitative analysis.

For cell viability assay, in a comparable manner [25], a cell suspension of 1 × 10^5^ cells/mL was seeded into a 96‐well plate. Following a 24 h incubation period, complete medium supplemented with 0.01% SDS was added to induce cellular damage. Concurrently, 0.75 μg/mL WY14643 was used as a positive control. After an additional 24 h, cell viability was evaluated using the CCK8 assay to determine the extent of cellular damage. Each experimental group comprised six replicate wells.

#### 2.5.6 Validation of Anti‐Inflammatory Property

In vitro anti‐inflammatory investigations employed classic lipopolysaccharide (LPS; Merck KGaA, Darmstadt, Germany) to elicit an inflammatory response in RAW 264.7 cells [26]. To evaluate the anti‐inflammatory properties of PGC, 5 μg/mL quercetin (Macklin Biochemical Technology) was used as a positive control. After exposure of RAW 264.7 cells to 100 ng/mL LPS for 24 h, the concentration of NO in the supernatant of each experimental group was quantified using an NO detection kit (Beyotime Biotechnology) to determine the extent of cellular inflammation. Each experimental group consisted of six replicate wells, and the OD of each well was measured at 530 nm. A standard curve correlating OD values with known NO concentrations was generated to obtain the concentration in the test samples.

#### 2.5.7 Validation of Oil Control Property

Nile red was used as a staining agent for intracellular lipid droplets to assess the capacity of cells to synthesize lipids [27]. SZ95 cells were inoculated at a density of 1 × 10^5^ cells/mL into a 96‐well plate and incubated with complete medium for 24 h. An equivalent volume of complete medium containing dihydrotestosterone (DHT; Solarbio Life Sciences) at a concentration of 6 μg/mL and palmitic acid (PA; Kunchuang Biotechnology, Xian, China) at 500 μg/mL was added to enhance the process of adipogenesis; 0.25% panthenol (Aladdin Scientific) was added to one well, which served as the positive control. Each treatment group received a complete medium containing DHT and PA along with the respective PGC doses. All groups were incubated under standard culture conditions for 48 h. Following incubation, the supernatant was discarded, and the cells were washed twice with PBS. Subsequently, 200 μL of 4% paraformaldehyde fixative (Yongjin Biotechnology, Guangzhou, China) was added to each well for fixation for 30 min. Following fixation, the fixative was removed, and the cells were washed twice with PBS. Next, 0.5% Triton X‐100 (Aladdin Scientific) was added, and the cells were incubated in the dark for 5 min. After washing twice with PBS, 10 μg/mL Nile Red dye (Aladdin Scientific) was added, and the cells were incubated in the dark for an additional 10 min; DAPI was used to visualize the cell nuclei. Fluorescence images were captured under a fluorescence microscope, and the average fluorescence intensity for each field of view was quantified using the Image J software. Each group was analyzed across six fields of view.

## 3 Results

### 3.1 Stability Assessment and Active Substances Detection

**FIGURE 2:**
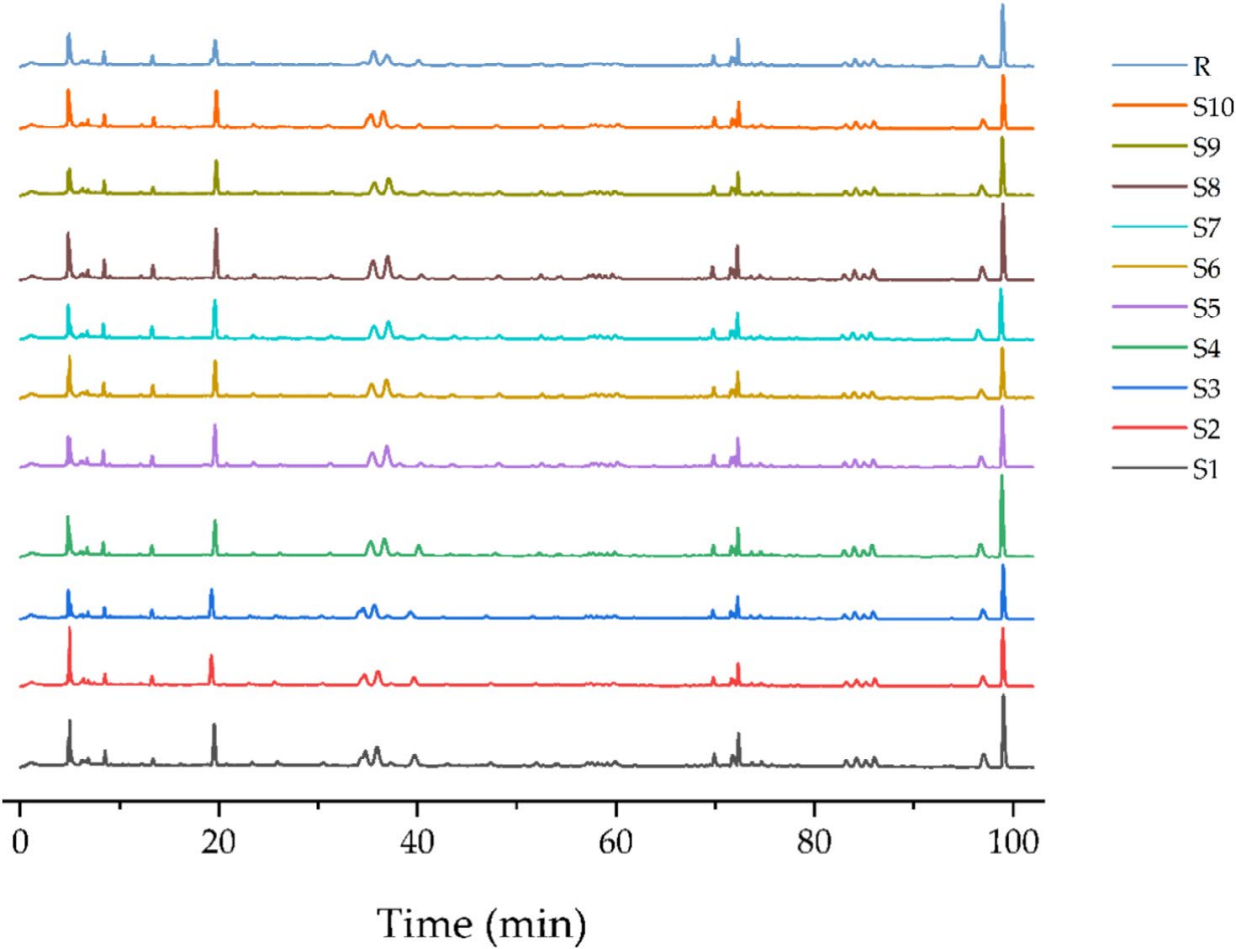
The fingerprints for the 10 PGC batches.

Given the inherent characteristics of the combined extract, verification of the stability of the composition and assessment of the similarities among various batches is essential [28]. We analyzed the liquid chromatograms from 10 distinct PGC batches using the “Similarity Evaluation System for Chromatographic Fingerprint of Traditional Chinese Medicine (2012).” Using S1 as the reference spectrum, we manually aligned the chromatographic fingerprints of the 10 batches, resulting in the generation of a control fingerprint spectrum, designated R. The HPLC fingerprint spectrum of PGC is shown in Figure 2. We subjected 10 PGC batches to similarity assessment. Based on the control fingerprint spectrum R as the benchmark, we determined the similarity indices for each PGC batch with all values ≥ 0.9 (Table 2). We measured the total phenolic acid, flavonoid and alkaloid contents, equivalent to chlorogenic acid, quercetin and berberine, respectively, using an ultraviolet spectrophotometer. We detected a considerable amount of potentially biologically active substances such as phenolic acids (5.17 ± 0.457 mg CE/mL), flavonoids (9.44 ± 0.241 mg QE/mL) and alkaloids (1.18 ± 0.122 mg BE/mL) in PGC (Table 2).

**TABLE 2:** The similarity evaluation and biologically active substance contents among the 10 batches.

No.	Batch	Similarity	Biologically active compounds		
			TPC (mg CE/mL)	TFC (mg QE/mL)	TAC (mg BE/mL)
S01	20240810‐32	0.991	5.05 ± 0.026	9.41 ± 0.004	1.35 ± 0.004
S02	20240812‐33	0.997	5.11 ± 0.021	9.10 ± 0.014	1.18 ± 0.003
S03	20240814‐34	0.995	5.57 ± 0.005	9.87 ± 0.067	1.25 ± 0.005
S04	20240815‐35	0.994	4.62 ± 0.027	9.15 ± 0.032	1.36 ± 0.005
S05	20240816‐36	0.998	5.87 ± 0.004	9.60 ± 0.034	1.00 ± 0.001
S06	20240817‐37	0.996	5.81 ± 0.094	9.49 ± 0.034	1.07 ± 0.003
S07	20240819‐38	0.997	5.33 ± 0.003	9.67 ± 0.006	1.15 ± 0.003
S08	20240820‐39	0.998	5.14 ± 0.002	9.15 ± 0.006	1.22 ± 0.002
S09	20240821‐40	0.996	4.64 ± 0.005	9.52 ± 0.038	1.21 ± 0.001
S10	20240824‐42	0.993	4.60 ± 0.004	9.50 ± 0.013	1.00 ± 0.005
AVE	—	—	5.17 ± 0.457	9.44 ± 0.241	1.18 ± 0.122

Abbreviations: BE, berberine equivalent; CE, chlorogenic acid equivalent; QE, quercetin equivalent; TAC, total alkaloids content; TFC, total flavonoids content; TPC, total phenolic acids content.

### 3.2 Network Pharmacology and Molecular Docking Analysis

#### 3.2.1 Identification of Specific Components and Targets

**FIGURE 3:**
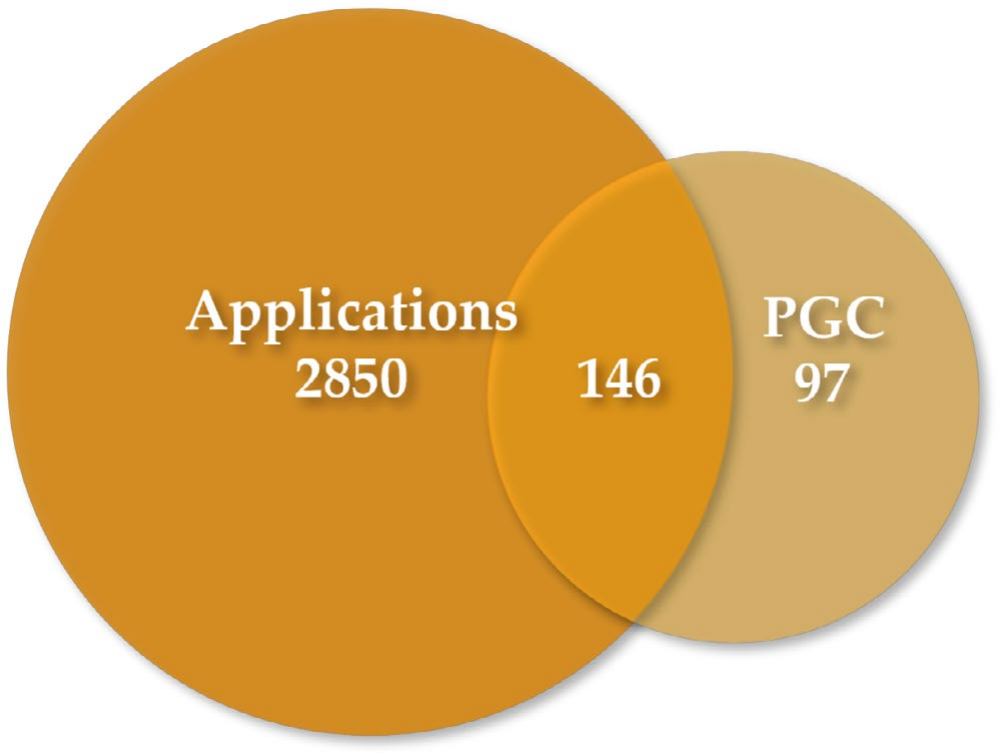
Venn diagram of PGC targets and application associated targets.

We aimed to ascertain the active constituents of each herb within the combined extract, resulting in the identification of 64 unique chemical compounds (with a bioavailability ≥ 30% and a drug‐likeness score ≥ 0.18) sourced from various databases. A total of 243 distinct and effective target genes corresponded to the active constituents. We extracted a comprehensive dataset comprising 2996 targets relevant to the intended cosmetics application from various databases. We also conducted an intersection analysis between the 243 selected targets associated with the active components of the combined extract and the application targets. This analysis was visually represented through a Venn diagram, which revealed 146 common targets shared by the active components in both PGC and applications (Figure 3).

#### 3.2.2 Construction of a “PGC‐Components‐Targets‐Applications” Network

**FIGURE 4:**
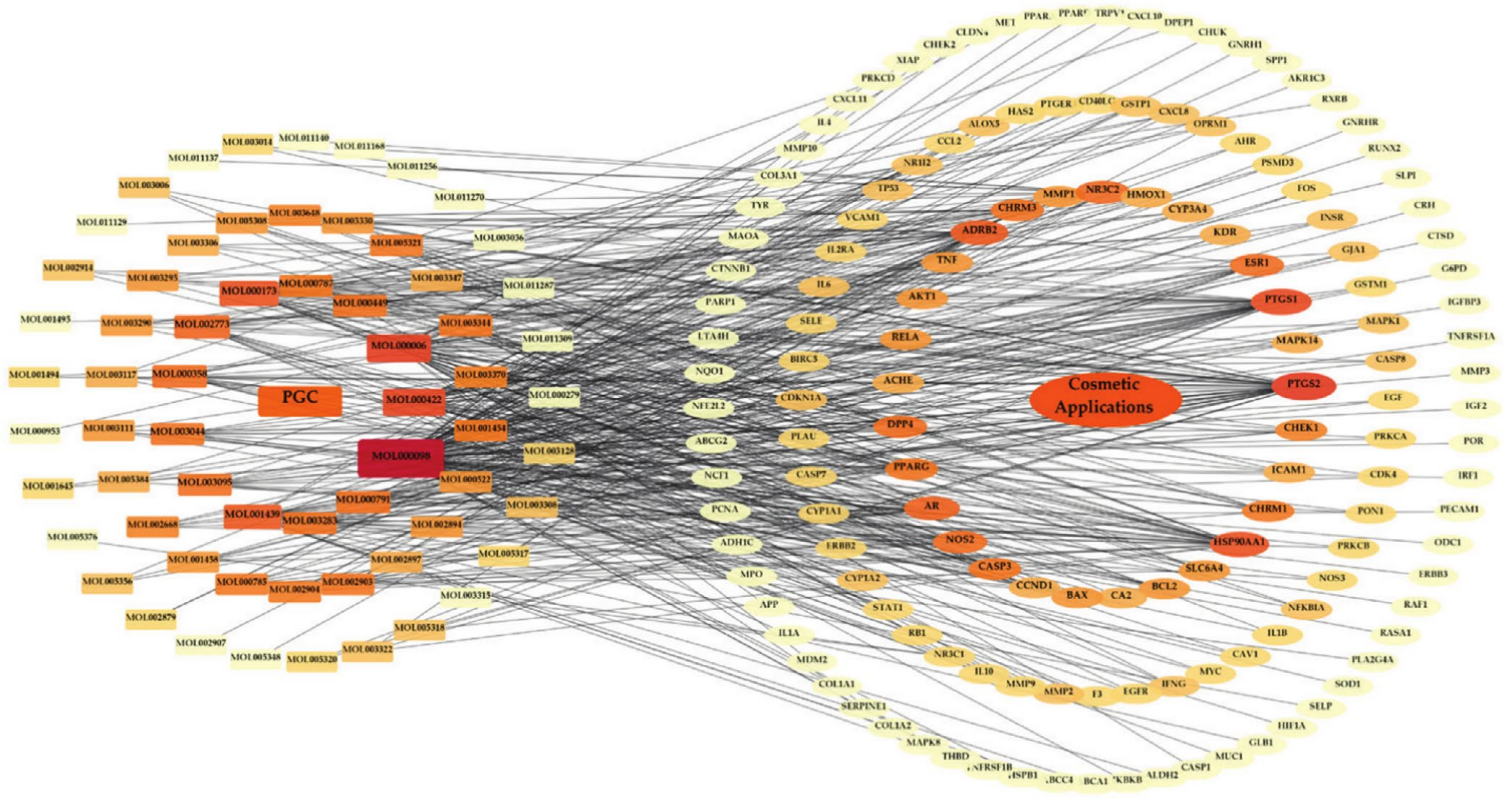
The “PGC‐Components‐Targets‐Applications” network diagram. Round rectangles denote the active compounds, whereas ellipse nodes indicate targets. The size of nodes is associated with their respective degree value.

Following the importation of 64 components and 146 associated targets into Cytoscape version 3.10.2, a “PGC‐Components‐Targets‐Applications” network was established, comprising 212 nodes and 486 edges (Figure 4). The nodes were systematically ranked from the inner to the outer circle, arranged in shapes that varied in size from large to small and in color from dark to light red, reflecting their degree of connectivity from high to low. Notably, quercetin (MOL000098), luteolin (MOL000006), kaempferol (MOL000422), wogonin (MOL000173), arachidonic acid (MOL001439), beta‐carotene (MOL002773), beta‐sitosterol (MOL000358), 5‐hydroxy‐7‐methoxy‐2‐(3,4,5‐trimethoxyphenyl) chromone (MOL003095), bicuculline (MOL0000791), and chryseriol (MOL003044) exhibited higher degrees of connectivity than the other components.

#### 3.2.3 Construction of PPI Network

**FIGURE 5:**
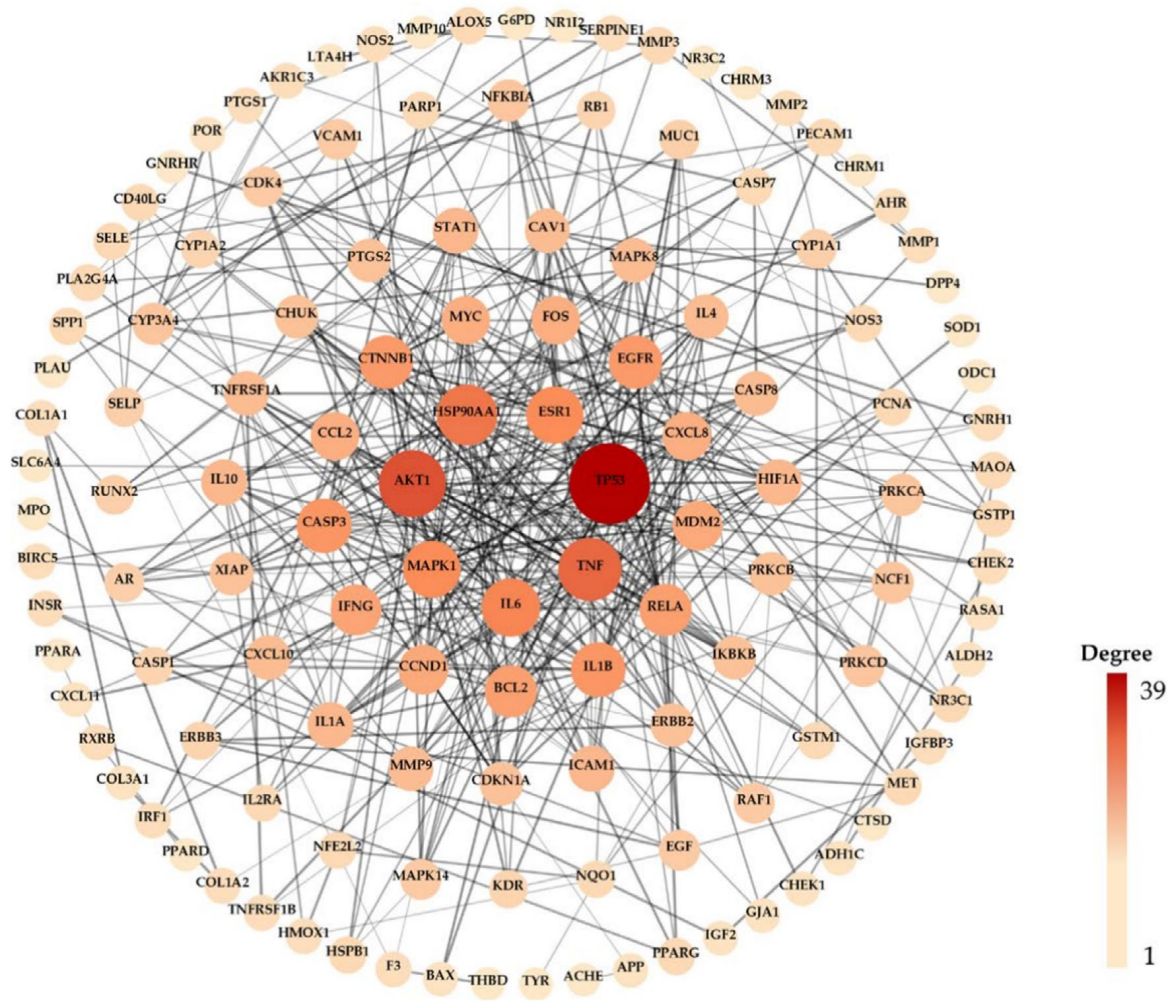
The PPI network of hub targets.

After importing the targets into the STRING database, 130 nodes were preserved within the network, with 874 edges connecting the nodes (Figure 5). The nodes were organized in a circular arrangement, with their positioning reflecting their degree of connectivity, ranging from high to low, represented by ellipses that varied in size from large to small and in color from dark to light red. Similarly, the edges were ranked according to their combined scores, with thicker lines indicating higher scores and thinner lines indicating lower scores. Notably, 16 nodes (TP53, AKT1, TNF, HSP90AA1, IL6, ESR1, MAPK1, CASP3, IL1B, CTNNB1, EGFR, BCL2, RELA, IFNG, CCND1, and MDM2) exhibited a greater degree of interaction compared with that of the other targets.

#### 3.2.4 Enrichment Analysis of GO and KEGG Pathway

**FIGURE 6:**
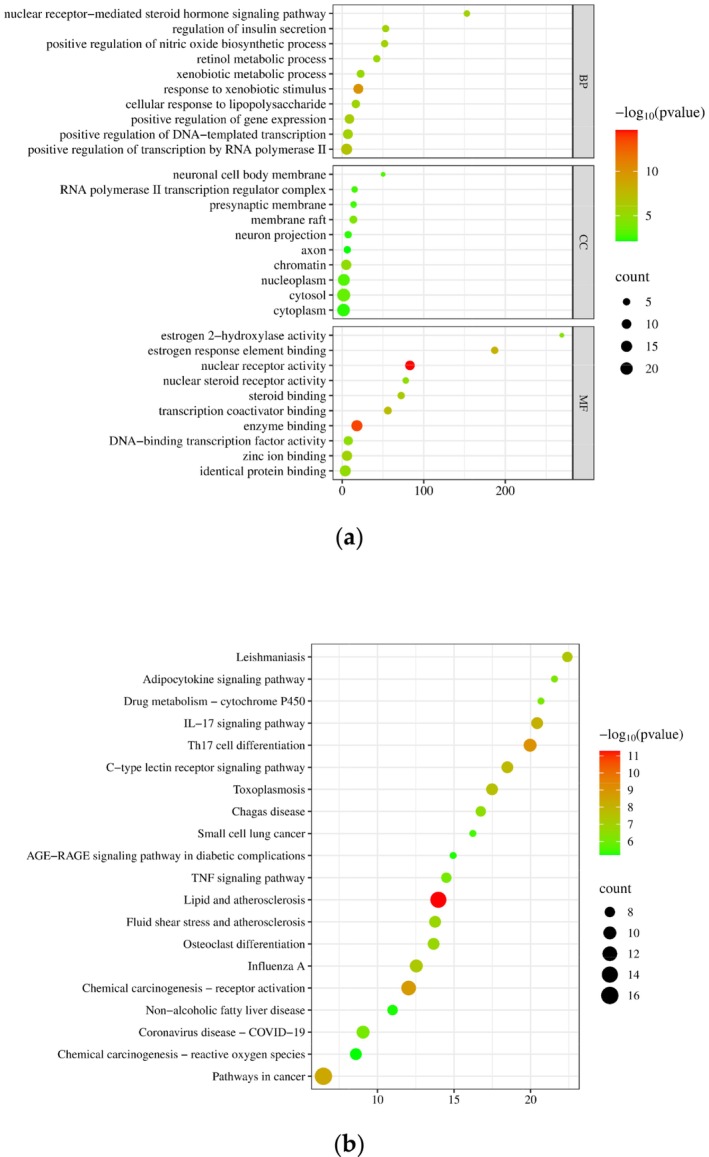
Bubble charts of (a) GO term enrichment analysis and (b) KEGG pathway enrichment analysis.

We conducted GO term enrichment analysis on the overlapping targets using the DAVID database, resulting in the identification of 166 biological processes, 20 cellular components, and 54 molecular functions, with a significance threshold of *p* < 0.05 (Figure 6a). Among these, the 10 most significant terms for BP, CC, and MF are presented below. The top 10 molecular function terms predominantly encompassed activities related to nuclear receptor function, enzyme binding, binding to estrogen response elements, transcription coactivator interactions, steroid binding, zinc ion binding, identical protein binding, nuclear steroid receptor activity, DNA‐binding transcription factor activity, and estrogen 2‐hydroxylase activity. In terms of cellular components, the leading 10 terms were primarily associated with chromatin, membrane rafts, the cytosol, nucleoplasm, neuronal cell body membranes, RNA polymerase II transcription regulator complexes, presynaptic membranes, neuron projections, the cytoplasm, and axons. Finally, the 10 most significant biological process terms were largely related to responses to xenobiotic stimuli, positive regulation of transcription by RNA polymerase II, enhancement of gene expression, positive regulation of DNA‐templated transcription, nuclear receptor‐mediated steroid hormone signaling pathways, regulation of insulin secretion, positive regulation of nitric oxide biosynthetic processes, cellular responses to lipopolysaccharide, retinol metabolic processes, and xenobiotic metabolic processes.

The results of the KEGG pathway enrichment analysis indicated that pathways in cancer exhibited the highest count number of associated gene targets, totaling 16 (Figure 6b). In addition, the lipid and atherosclerotic pathways were characterized by the lowest *p*‐value, signifying their status as the most statistically significant pathways. This pathway encompasses several gene targets including MMP1, PRKCA, TNF, RELA, ICAM1, NFKBIA, IKBKB, MAPK8, RXRA, IL1B, CASP1, CYP1A1, PPARG, and RXR.

#### 3.2.5 Analysis of Interactions Between PGC and Its Targets Based on Molecular Docking

**FIGURE 7:**
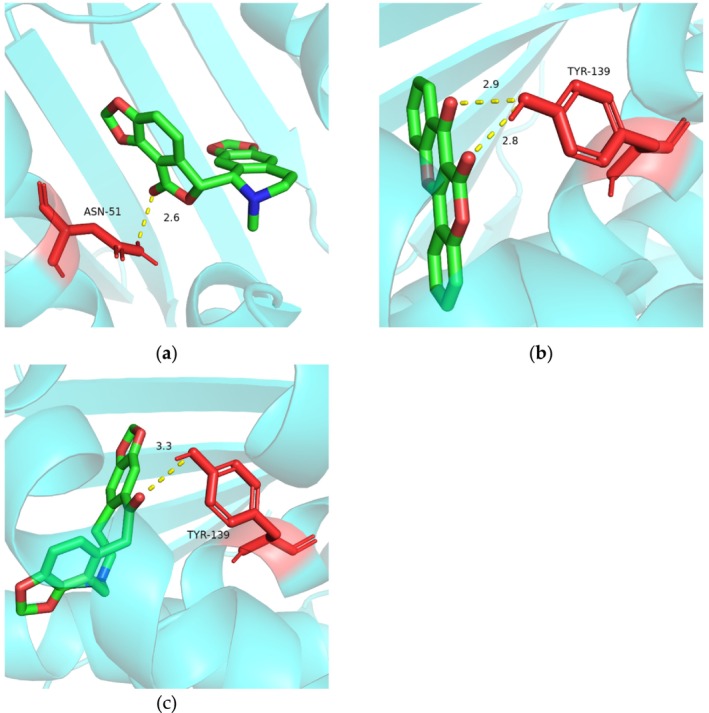
Molecular docking visualization (a) Potential binding position between bicuculline and HSP90AA1. (b) Potential binding position between frutinone A and HSP90AA1. (c) Potential binding position between fumarine and HSP90AA1.

To validate the interactions between the components of the combined extract and targets associated with specific applications, we analyzed the components and their corresponding overlapping targets to identify potential binding positions and energies. Typically, a binding energy value of ≤ −6 kcal/mol is indicative of strong binding activity. Approximately 89% of docking results demonstrated that the PGC components exhibited great binding activity (< −6 kcal/mol) with their respective targets (Table 3). Furthermore, the specific findings revealed that AKT1 and HSP90AA1 exhibited superior binding activity with certain components in PGC (< −10 kcal/mol). We observed an absence of hydrogen bonding between AKT1 and beta‐carotene in the visualization; however, a binding energy of ≤ −12 kcal/mol suggested a significant level of binding affinity between them. The primary interaction force was ascribed to the hydrogen bond formed between bicuculline and the residue (ASN‐51) of HSP90AA1 (Figure 7a). Additionally, two hydrogen bonds were established between frutinone A and residue (TYR‐139) of HSP90AA1 (Figure 7b), and a hydrogen bond was also present between fumarine and residue (TYR‐139) of HSP90AA1 (Figure 7c).

**TABLE 3:** The bonding energies between the components and their unique targets.

Ligand	Mol ID	Affinity (kcal/mol)
AKT1	MOL002773	−12
	MOL000006	−9.4
	MOL000173	−9.3
	MOL000422	−9.2
	MOL000098	−9.1
BCL2	MOL002773	−8.1
	MOL000173	−6.6
	MOL000358	−6.6
	MOL000098	−6.5
	MOL000422	−6.4
CASP3	MOL000006	−8.1
	MOL002773	−8.0
	MOL005344	−8.0
	MOL000098	−7.7
	MOL000422	−7.7
	MOL000173	−6.8
	MOL000358	−6.2
	MOL001439	−5.0
CCND1	MOL000173	−7.8
	MOL002903	−7.5
	MOL000098	−7.4
	MOL000006	−7.4
	MOL001439	−5.3
CTNNB1	MOL002773	−7.4
EGFR	MOL000006	−8.0
	MOL000098	−7.7
ESR1	MOL002668	−8.7
	MOL001458	−8.5
	MOL003044	−8.4
	MOL000173	−8.2
	MOL002897	−8.2
	MOL002894	−8.0
	MOL003290	−8.0
	MOL001454	−7.6
	MOL000785	−7.4
	MOL003283	−7.4
	MOL003095	−6.9
	MOL003111	−6.4
	MOL003014	−5.6
HSP90AA1	MOL000791	−10.8
	MOL005321	−10.7
	MOL000787	−10.4
	MOL003306	−9.9
	MOL002904	−9.8
	MOL003648	−9.7
	MOL000358	−9.6
	MOL001454	−9.6
	MOL000006	−9.3
	MOL000422	−9.3
	MOL000522	−9.3
	MOL005384	−9.3
	MOL002903	−9.2
	MOL003044	−9.2
	MOL000098	−9.1
	MOL000173	−9.0
	MOL003095	−9.0
	MOL003290	−8.8
	MOL000785	−8.6
	MOL003322	−8.6
	MOL003330	−8.5
	MOL005318	−8.5
	MOL002914	−7.9
	MOL003370	−7.9
	MOL003295	−7.8
	MOL003283	−7.5
	MOL003308	−7.5
	MOL003111	−7.2
IFNG	MOL000006	−7.3
	MOL000098	−7.2
	MOL005344	−7.1
IL1B	MOL000098	−7.4
	MOL005344	−7.1
IL6	MOL000006	−7.1
	MOL000098	−6.9
	MOL000173	−6.3
MAPK1	MOL000006	−7.7
	MOL000098	−7.6
	MOL001439	−4.1
MDM2	MOL000006	−7.0
RELA	MOL000173	−4.9
	MOL000006	−4.8
	MOL000098	−4.7
	MOL000422	−4.6
	MOL001439	−2.8
TNF	MOL005344	−6.8
	MOL000098	−6.3
	MOL000006	−6.1
	MOL000173	−6.1
	MOL000422	−6.0
TP53	MOL000006	−7.0
	MOL000098	−6.9
	MOL000173	−6.2

### 3.3 PGC Improves Cell Viability

**FIGURE 8:**
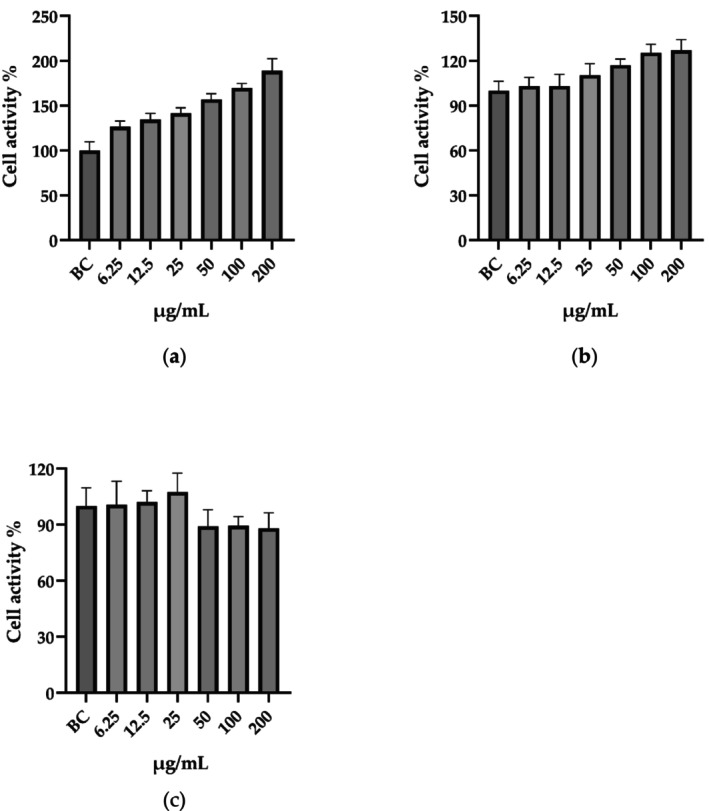
The effect of varying PGC concentrations on cellular activity. (a) HaCaT. (b) RAW 264.7. (c) SZ95.

Validate not only the safety of this combined extract through detailed experiments involving relevant cell lines, but also the determination of the appropriate safe dosage to conduct in vitro efficacy tests, is essential. We performed viability assays using the CCK8 kit. We found that PGC was safe at the experimental dosage for the HaCaT, RAW 264.7, and SZ95 cell lines (Figure 8a–c). Furthermore, PGC notably enhanced HaCaT cell proliferation, suggesting that this combination may have potential applications in skin repair.

### 3.4 PGC Actively Improved the Skin Condition

#### 3.4.1 PGC Optimized the Skin Structure

Exposure of the skin to highly irritating test substances or ultraviolet (UV) radiation can result in acute clinical damage to the skin barrier, manifesting as dryness and erythema. The anionic surfactant SDS can compromise the integrity of the skin barrier when applied at elevated concentrations [29]. Dysfunction of the skin barrier may elevate the susceptibility to allergic conditions, including atopic dermatitis [30]. HE staining serves as an indicator of alterations in the skin barrier under various conditions. For example, following damage induced by SDS, the skin barrier typically exhibits increased looseness and reduced thickness of the viable cell layer. LOR is a critical component in the formation of the keratinized envelope, constituting approximately 80% of its composition and playing a significant role in structural reinforcement [31]. A decrease in LOR protein levels is the primary contributor to impaired skin barrier function. FLG is another essential component in this context [32]. Therefore, the efficacy of the test sample under investigation can be assessed by examining the morphological changes in the tissue of the three‐dimensional skin model, as well as the levels of LOR and FLG.

**FIGURE 9:**
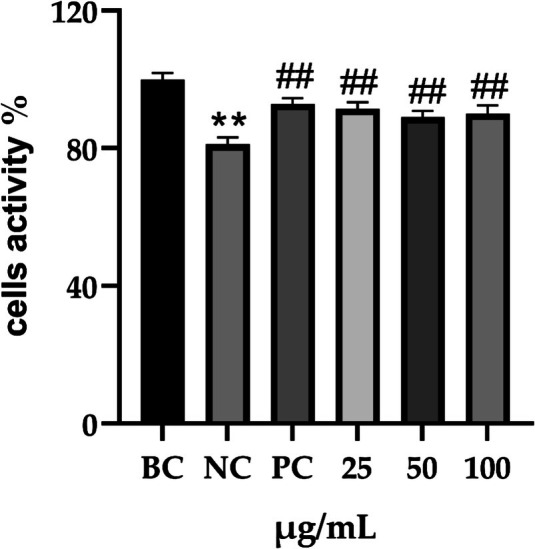
Effect of PGC on cell activity after SDS stimulation. Each bar represents the mean ± SD of study (*n* = 6); versus BC, p** < 0.01; versus NC, *p* ^##^ < 0.01 when compared between two groups (Student's *t*‐test).

HaCaT cells were simultaneously treated with SDS and PGC for 24 h, after which cell viability was evaluated. We observed a significant reduction in cell viability after 24 h of SDS treatment compared with that in the control group (*p* < 0.001, 95% CI: 17.64–20.14). Furthermore, the enhancement in cell viability observed after PGC treatment was comparable to that achieved with the positive control drug, WY14643, a peroxisome proliferator capable of activating particular peroxidases and the proliferator‐activated receptor. The viability of cells treated with either PGC or WY14643 was significantly higher than that observed in the SDS‐treated group (PC: *p* < 0.001, 95% CI: 9.251–14.23; 25 μg/mL: *p* < 0.001, 95% CI: 8.206–12.43; 50 μg/mL: *p* < 0.001, 95% CI: 5.512–10.30; 100 μg/mL: *p* < 0.001, 95% CI: 5.875–11.95; Figure 9).

**FIGURE 10:**
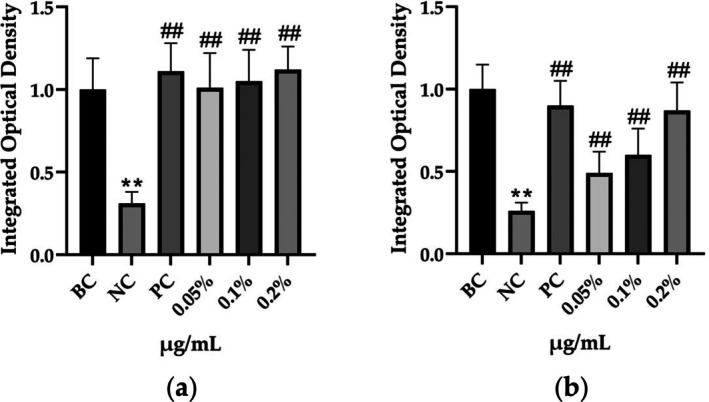
Effects of PGC on FLG and LOR expression. (a) Integrated optical density of LOR. (b) Integrated optical density of FLG. Each bar represents the mean ± SD of study (*n* = 6); versus BC, p** < 0.01; versus NC, *p* ^##^ < 0.01 when compared between two groups (Student's *t*‐test).

The morphological changes identified by HE staining indicated that the living cell layer of the epidermal model in the NC group exhibited damage and vacuoles. In contrast to the NC group, the living cell layer of the epidermal model in the PC group displayed a more compact arrangement, fewer vacuoles, and significant improvement in living cell integrity. This improvement was evident in the groups treated with PGC at concentrations of 0.1% and 0.2%, whereas the group treated with 0.05% PGC exhibited comparatively less improvement (Table 4). Immunofluorescence analysis revealed a significant reduction in the LOR (*p* < 0.001, 95% CI: 0.562–0.817) and FLG (*p* < 0.001, 95% CI: 0.644–0.835) protein levels in the NC group (Table 5). Following WY14643 administration, a significant increase occurred in the protein levels of LOR (*p* < 0.001, 95% CI: 0.668–0.930) and FLG (*p* < 0.001, 95% CI: 0.538–0.733). Furthermore, all PGC treatment groups also demonstrated significant increases in LOR (0.05%: *p* < 0.001, 95% CI: 0.571–0.824; 0.1%: *p* < 0.001, 95% CI: 0.618–0.870; 0.2%: *p* < 0.001, 95% CI: 0.699–0.923) and FLG (0.05%: *p* < 0.001, 95% CI: 0.129–0.325; 0.1%: *p* < 0.001, 95% CI: 0.244–0.436; 0.2%: *p* < 0.001, 95% CI: 0.514–0.706) levels. Additionally, the maximum enhancement in the levels of LOR and FLG proteins reached 261.29% and 234.62%, respectively, in 0.2% PGC, exhibiting a dose‐dependent relationship with the expression of proteins (Figure 10).

**TABLE 4:** Effect of PGC on the pathological changes of 3D skin model (HE staining).

Group	200×	Group	200×
BC	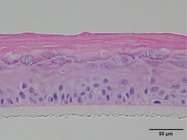	NC	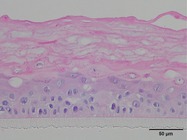
PC	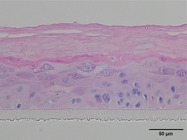	0.05%	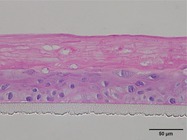
0.1%	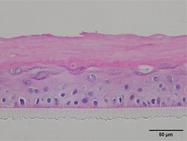	0.2%	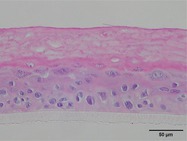

*Note:* HE staining of the 3D skin model, in which cell nuclei are blue and cytoplasms are purple‐red.

**TABLE 5:** Results of FLG and LOR protein immunofluorescence staining.

Type	Group	200×	Group	200×
LOR	BC	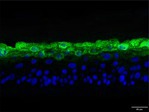	NC	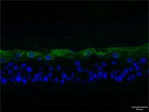
	PC	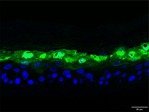	0.05%	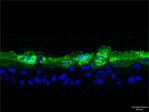
	0.1%	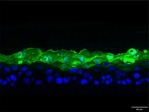	0.2%	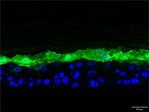
FLG	BC	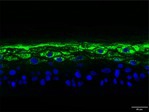	NC	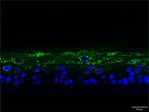
	PC	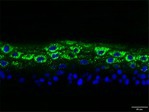	0.05%	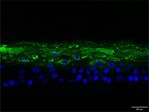
	0.1%	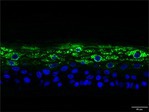	0.2%	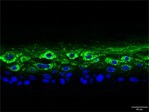

*Note:* Immunofluorescence detection in the 3D skin model with cell nuclei in blue and unique proteins in green.

#### 3.4.2 PGC Promoted Cell Migration

**FIGURE 11:**
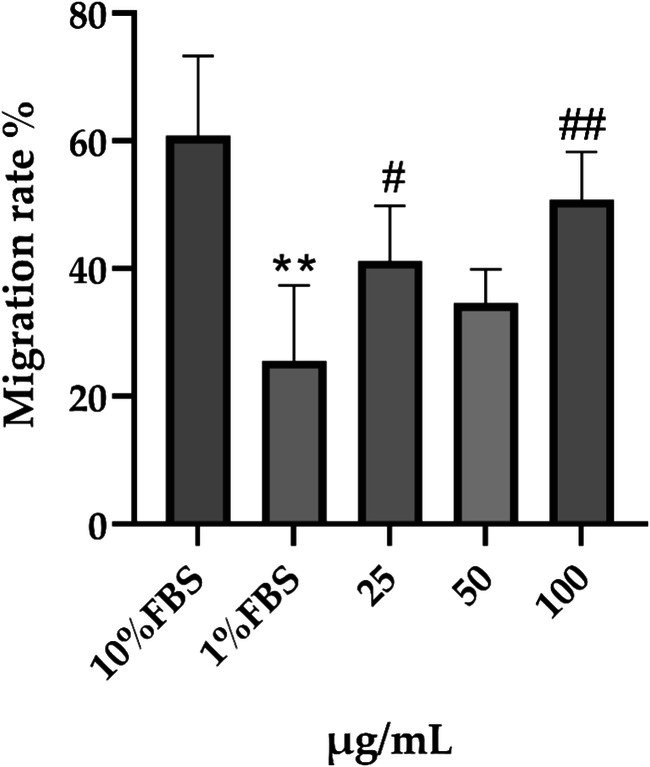
Effect of PGC on migration rate. Each bar represents the mean ± SD of study (*n* = 6), versus 1% FBS, p** < 0.01; versus 10% FBS, 0.01 < *p* ^#^ < 0.05, *p* ^##^ < 0.01 when compared between two groups (Student's *t*‐test).

Skin repair is a multifaceted biological process that primarily depends on the interactions among keratinocytes, fibroblasts, and associated cytokines to facilitate the re‐epithelialization of injured tissue. Keratinocytes are pivotal in this process, as they contribute to the repair and coverage of the wound surface through various biological activities, including proliferation and migration, thereby playing a dominant role in the re‐epithelialization of the affected area. Concurrently, these cells secrete a range of cytokines and growth factors that collectively regulate and enhance the wound‐healing process. In the context of a cell scratch assay, systematic measurement and comparison of scratch width or area serve as an effective means to assess cell migration capabilities. After a 16‐h period postscratching, the migratory behavior of the cells significantly reduced the scratch area (Table 6). We observed that the migration rate in the 10% FBS group was markedly higher than that in the 1% FBS group (*p* < 0.001, 95% CI: 27.89–42.50). Furthermore, nearly all PGC treatment groups exhibited superior repair capabilities compared with the 1% FBS group, with significant differences noted between the PGC groups at concentrations of 25 and 100 μg/mL and the 1% FBS group (25 μg/mL: *p* = 0.018, 95% CI: 0.4687–30.81; 100 μg/mL: *p* < 0.001, 95% CI: 15.16–35.29; Figure 11).

**TABLE 6:** Cell migration in each group within 16 h.

Groups	0 h	16 h
1% FBS	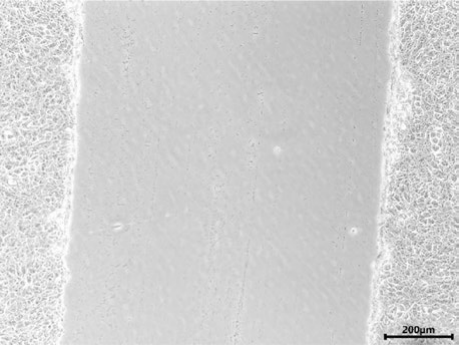	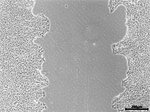
10% FBS	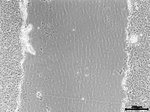	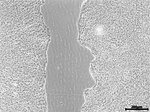
25 μg/mL	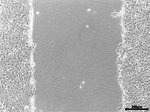	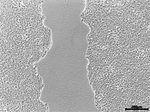
50 μg/mL	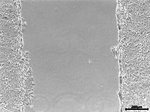	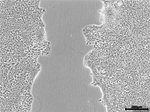
100 μg/mL	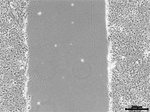	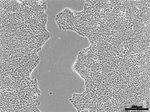

### 3.5 PGC Eliminated Acne

#### 3.5.1 PGC Inhibited Acne Related Bacteria

Using the microdilution method, we assessed the inhibitory effects of PGC on the growth of acne‐associated *Staphylococcus aureus* , *Cutibacterium acnes*, and *Staphylococcus epidermidis* . As indicated in Table 7, the MICs of PGC for *Staphylococcus aureus* , *Cutibacterium acnes*, and *Staphylococcus epidermidis* were 50, 25, and 25 μg/mL, respectively. In accordance with the antimicrobial activity classification of plant extracts established by The Clinical and Laboratory Standards Institute (CLSI) [33], the MIC of PGC falls below the threshold of 500 μg/mL, thereby demonstrating its high antimicrobial activity (Table 7).

**TABLE 7:** MIC of PGC against bacteria.

	MIC (μg/mL)	
	PGC	Penicillin V potassium
*Staphylococcus aureus*	50	2
*Cutibacterium acnes*	25	1
*Staphylococcus epidermidis*	25	1

#### 3.5.2 PGC Inhibited Adipogenesis of SZ95

**FIGURE 12:**
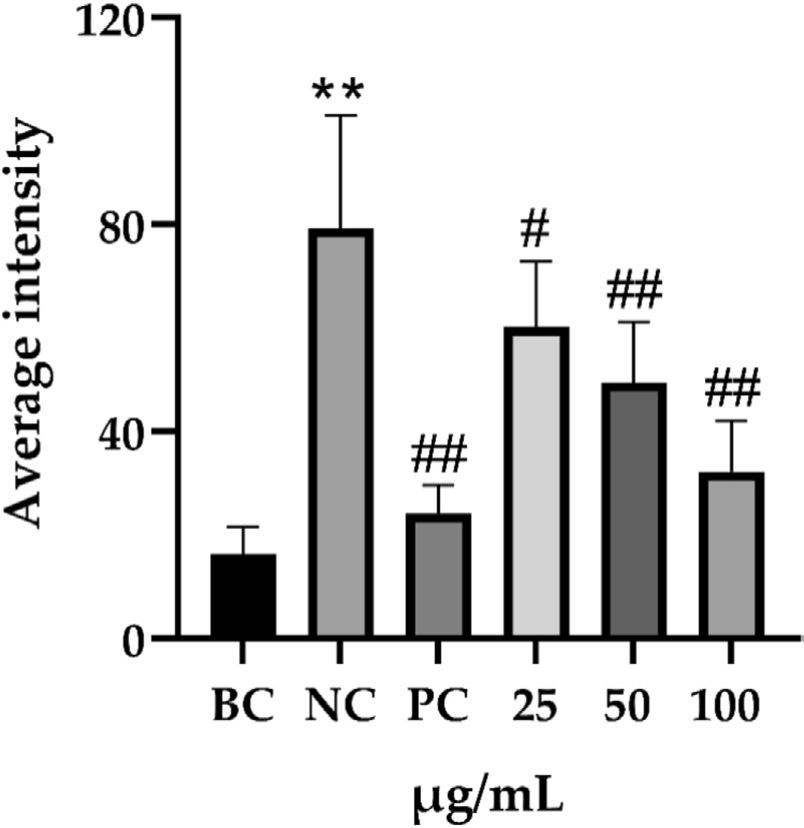
Oil control properties of PGC after DHT and PA induction. Each bar represents the mean ± SD of study (*n* = 6), versus BC, p** < 0.01; versus NC, 0.01 < *p* ^#^ < 0.05, *p* ^##^ < 0.01 when compared between two groups (Student's *t*‐test).

Endogenous lipids are secreted by sebaceous gland cells and subsequently enter hair follicles via their ducts. A portion of these lipids is released through the skin pores, forming a sebum film in conjunction with water that moisturizes the skin and maintains its barrier integrity. Conversely, excessive lipid secretion can lead to pore obstruction, contributing to acne development. In the present study, excessive lipid production was induced in SZ95 cells by DHT and PA treatment. Lipid accumulation was quantified using Nile Red, a lipid‐specific fluorescent dye, under a fluorescence microscope (Table 8). We found that DHT and PA treatment resulted in significantly higher levels of red fluorescence than in the blank control group (*p* < 0.001, 95% CI: 40.50–85.53). As anticipated, all groups treated with PGC or the positive control drug demonstrated a reduction in red fluorescence intensity, suggesting that PGC effectively inhibited lipid production. Statistical analysis of the average fluorescence intensity across all experimental groups revealed significant differences among PGC groups and the DHT and PA group (PC: *p* < 0.001, 95% CI: 27.82–82.47; 25 μg/mL: *p* = 0.047, 95% CI: 3.671–40.97; 50 μg/mL: *p* = 0.007, 95% CI: 7.911–51.52; 100 μg/mL: *p* < 0.001, 95% CI: 16.08–77.98; Figure 12).

**TABLE 8:** Variations in fluorescence intensity of the Nile Red probes.

Groups	Nile Red/DAPI	Groups	Nile Red/DAPI
BC	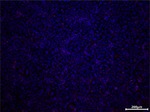	NC	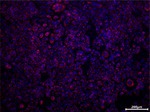
PC	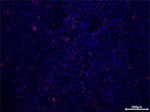	25 μg/mL	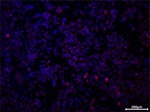
50 μg/mL	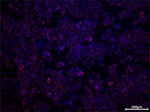	100 μg/mL	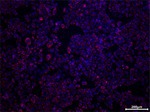

*Note:* Nile Red fluorescent probe detection in SZ95 cells, with cell nuclei in blue and lipid droplets in red.

#### 3.5.3 PGC Inhibited LPS‐Induced Inflammation

**FIGURE 13:**
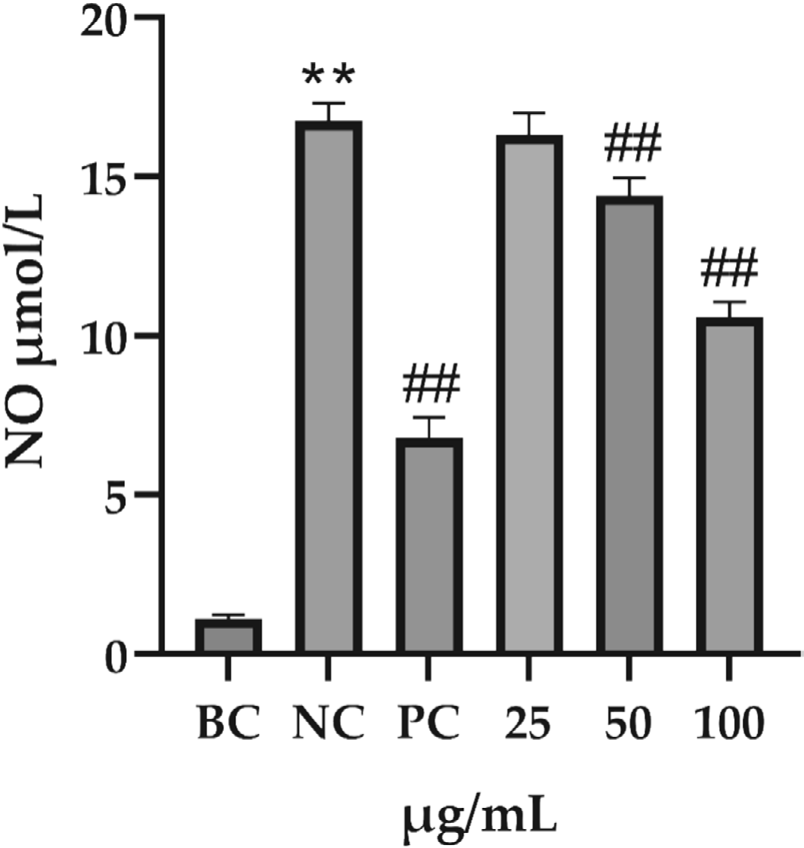
Variations in NO levels within the supernatant of cell cultures. Each bar represents the mean ± SD of study (*n* = 6), versus BC, p** < 0.01; versus NC, *p* ^##^ < 0.01 when compared between two groups (Student's *t*‐test).

To investigate the beneficial effects of PGC on inflammation, we conducted an assay using LPS to induce inflammation in RAW 264.7 cells. The concentration of NO in the cell supernatant served as an indicator of the activity of inducible nitric oxide synthase (iNOS), which was activated during the inflammatory response. Specifically, LPS at a concentration of 100 ng/mL resulted in a significant increase in NO levels (*p* < 0.001, 95% CI: 15.13–16.10), whereas PGC (50 and 100 μg/mL) and PC treatments significantly mitigated this increase (*p* < 0.001, 95% CI: 2.055–2.592; *p* < 0.001, 95% CI: 5.974–6.325; Figure 13). Additionally, we observed alterations in cell morphology; typically, RAW 264.7 cells exhibit a round or oval shape with a darker coloration; however, following LPS induction, they adopted a spindle‐shaped or elongated spindle‐shaped morphology. We observed two distinct morphologies in each PGC‐treated group (Table 9). These findings suggested that PGC possesses the ability to inhibit inflammation, demonstrating a dose‐dependent relationship with the severity of the inflammatory response.

**TABLE 9:** Morphological alterations of RAW 264.7 in each group following LPS induction.

Group	200×	Group	200×
BC	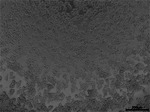	NC	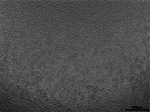
PC	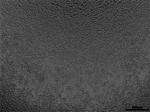	25 μg/mL	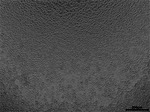
50 μg/mL	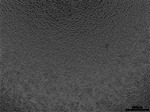	100 μg/mL	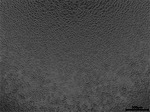

### 3.6 PGC Soothed Skin Allergy

**FIGURE 14:**
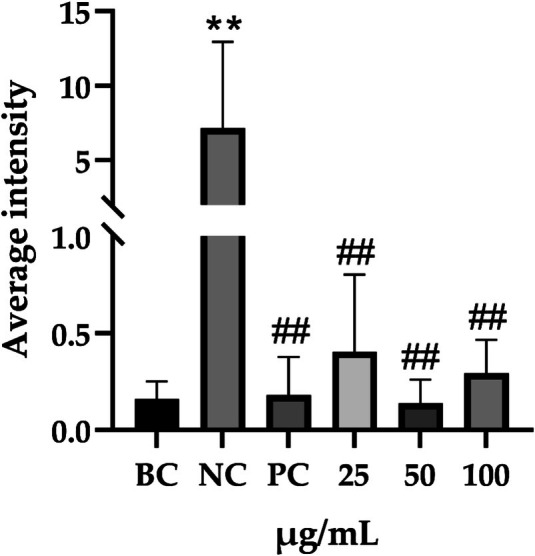
The antioxidant properties of PGCs after H_2_O_2_ stimulation. Each bar represents the mean ± SD of study (*n* = 6), versus BC, p** < 0.01; versus NC, *p* ^##^ < 0.01 when compared between two groups (Student's *t*‐test).

Regarding the cellular peroxidative damage induced by hydrogen peroxide, we observed that, following exposure to a specific concentration of hydrogen peroxide, the cells exhibited an increased average fluorescence intensity when assessed with fluorescent probes (*p* = 0.007, 95% CI: 2.291–11.73). Vitamin C was employed as a positive control as its antioxidant properties significantly reduce ROS generation (*p* = 0.007, 95% CI: 2.329–11.65). Similarly, PGC application resulted in a significant reduction in average fluorescence intensity (25 μg/mL: *p* = 0.009, 95% CI: 3.671–40.97; 50 μg/mL: *p* = 0.007, 95% CI: 7.911–51.52; 100 μg/mL: *p* = 0.008, 95% CI: 16.08–77.98; Table 10), thereby demonstrating that PGC can mitigate ROS generation, indicating its antioxidant properties (Figure 14).

**TABLE 10:** Variations in fluorescence intensity of the DCFH‐DA probe.

Groups	DCFH‐DA/DAPI	Groups	DCFH‐DA
BC	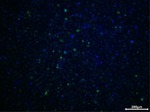	NC	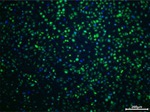
PC	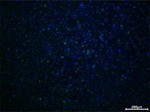	25 μg/mL	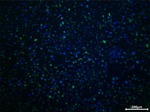
50 μg/mL	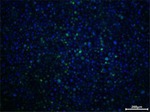	100 μg/mL	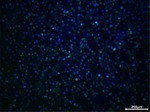

*Note:* DCFH‐DA fluorescent probe in HaCaT cells with cell nuclei in blue and specific probe in green.

## 4 Discussion

A complex interplay exists between humans and microorganisms that is particularly evident in the skin microbiome [34]. Research has indicated that alterations in this microbiome can disrupt this delicate balance, resulting in changes in the reactivity of the immune system and subsequently contributing to the development of inflammatory diseases [35, 36, 37]. Such conditions include atopic dermatitis, psoriasis, and acne, most of which are characterized by compromised skin barriers [38]. Given the multifactorial nature and diverse manifestations of skin issues, solutions must not address a singular factor or symptom. Instead, a “multitarget and multiefficacy” approach to formulation is required. The “multicomponent” properties of herbs align well with the current demands for diverse therapeutic solutions. To investigate the potential applications of PGC in the cosmetics industry, network pharmacology, molecular docking, and experimental validation were employed to demonstrate its efficacy in addressing various skin issues and their associated symptoms. In the network pharmacology phase, we preliminarily predicted the interactions and therapeutic effects of PGC on skin‐related conditions. Molecular docking results corroborated these effects and provided insights into the targets of PGC. Finally, experimental validation confirmed the findings derived from network pharmacology analysis. This study offers a comprehensive and cost‐effective elucidation of the fundamental principles underlying the application of PGC in cosmetics.

Comprehensive screening of databases yielded 64 chemical compositions associated with PGC, with 146 distinct and effective target genes corresponding to these active constituents. Furthermore, PPI analysis facilitated the identification of 16 hub targets, including cellular tumor antigen p53 (TP53), RAC‐alpha serine/threonine‐protein kinase (AKT1), and tumor necrosis factor. Subsequent GO and KEGG analyses further corroborated that PGC may influence Th17 cell differentiation and several signaling pathways, including the IL‐17, adipocytokine, TNF, and NF‐κB signaling pathways, thereby contributing to the amelioration of acne, atopic dermatitis, and skin barrier impairment. Additionally, the results of molecular docking studies reaffirmed the strong binding affinity between the active PGC compounds and the corresponding proteins. GO terms and KEGG pathway enrichment analyses of the PPI network indicated that PGC may influence apoptosis, cell cycle, inflammation, and adipocyte‐related signaling pathways, which appear to play regulatory roles in acne, atopic dermatitis, eczema, and skin barrier impairment. The most significant functional module within the PPI network was enriched with numerous genes that modulate inflammation, including hub targets such as TNF, IL6, and IL1B, along with several other targets implicated in the regulation of inflammatory responses in the body. These findings suggested that the modulation of cellular inflammation via hub targets may represent a critical mechanism by which PGC addresses various dermatological issues. Additionally, TP53, a pivotal tumor suppressor gene encoding the p53 protein, functions as a crucial transcription factor that regulates the expression of a multitude of target genes and mediates diverse cellular processes, including cell cycle progression and apoptosis. Previous studies have demonstrated that p53 influences several transcription factors associated with the pathogenesis of acne vulgaris, including FoxO1, androgen receptors, and essential genes involved in autophagy and apoptosis induction [39]. AKT1, also referred to as protein kinase B, is a vital component of cell signaling pathways that play a role in the regulation of various processes, including cell growth, survival, metabolism, and differentiation [40]. Modifications in phosphorylation of the AKT signaling pathway can promote epithelial wound healing [41]. Enhanced wound healing in tissue‐engineered human corneas was observed through altered phosphorylation of CREB and AKT signal transduction pathways. Subsequent investigations revealed that despite the use of different pharmacological agents targeting distinct acne pathogenesis mechanisms, a synergistic mode of action was observed, characterized by the attenuation of the AKT/mTORC1 signaling and enhancement of p53 signaling [42]. Collectively, these results provide a theoretical foundation and a reliable framework for future in vivo and in vitro experimental validation.

Based on the aforementioned studies, we conducted further experiments to assess the potential efficacy of PGC. We observed that PGC exerts a notable inhibitory effect on *Cutibacterium acnes*, the primary pathogen responsible for acne, *Staphylococcus aureus* , which is linked to atopic dermatitis, and *Staphylococcus epidermidis* , a commensal species of the skin microbiome. Notably, PGC demonstrated minimal cytotoxicity towards RAW 264.7, HaCaT, and SZ95 cell lines. Additionally, PGC effectively reduced NO levels in the supernatant of LPS‐stimulated RAW 264.7, thereby exhibiting a pronounced anti‐inflammatory effect. The results of our study are consistent with other studies demonstrating that lucidumol A and ganodermanontriol contained in *Ganoderma lucidum* can effectively reduce LPS‐induced RAW 264.7 inflammatory responses, and that ginsenoside Rg3 can inhibit iNOS activity [43, 44]. In oil control experiments utilizing SZ95 cells, PGC significantly diminished the secretion of lipid droplets. Previous research has demonstrated that ginsenoside F2 from *Panax ginseng* suppressed adipogenesis by regulating the expression of adipokines and activating the AMPK pathway [45], in consistency with our network pharmacology findings. Ergosterol peroxide from *Ganoderma lucidum* inhibited the expression of fatty acid synthase (FAS), fatty acid translocase (FAT), and acetyl‐coenzyme A carboxylase (ACC), which are lipogenic factors [46]. Furthermore, research has indicated that various ginsenosides can facilitate cell migration and enhance the integrity of the skin barrier, as demonstrated by both in vitro and in vivo animal studies [47, 48, 49]. In the present study, in vitro experiments utilizing HaCaT cells demonstrated improved cell viability following SDS‐induced damage and promoted cell migration in scratch assays, suggesting a reparative effect. Additionally, analysis of pathological sections from the 3D epidermal skin model corroborated that PGC improved the structural integrity of the compromised skin barrier. Immunofluorescence assays revealed that PGC led to an increase in the level of FLG and LOR, which are associated with skin barrier function.

Ultimately, PGC contributes to skin barrier restoration, thereby preventing the occurrence and recurrence of skin issues. The MIC of PGC against *Cutibacterium acnes* (25 μg/mL) was significantly lower than that of tea tree oil (MIC = 500 μg/mL) [50]. Moreover, its efficacy in suppressing NO production (40% reduction at 50 μg/mL) demonstrated a concentration advantage over glycyrrhizin (35% reduction at 100 μg/mL) [51]. This high efficacy might stem from its multi‐target mechanism of action: molecular docking revealed strong binding between β‐carotene and AKT1 (−12 kcal/mol), potentially regulating the AKT/mTORC1 pathway to inhibit sebum secretion, whereas the binding of quercetin to HSP90AA1 (−9.1 kcal/mol) might synergistically suppress the release of inflammatory factors. Compared with single‐component agents (e.g., benzoyl peroxide, which only exhibits antibacterial effects [52]), the multi‐efficacy of PGC enables it to simultaneously display antibacterial, anti‐inflammatory, and reparative functions, aligning with the current trend of “simplified formulations” in cosmetics [53].

This study had some limitations. The lack of clinical or in vivo research limits the translational relevance of PGC. The complexity of its composition may lead to variations in transdermal absorption, necessitating future optimization of delivery efficiency through nanocarrier technology [54]. Furthermore, this research is deficient in experimental evidence that would clarify the specific signaling pathways associated with skin issues that PGC may influence. Thus, further exploration is needed to validate the utility of PGC, a novel, combined herbal formulation.

## 5 Conclusion

Despite the limitations, our study revealed that PGC demonstrates significant potential for use in cosmetic applications owing to its “multicomponent” and “multitarget” characteristics. Specifically, by employing bioinformatics analysis, including network pharmacology and molecular docking, the potential efficacy and targets of PGC were predicted. The efficacy of PGC in addressing acne‐related skin dysfunctions was systematically assessed from multiple viewpoints: microbial hypercolonization (*C. acnes*), barrier compromise (SDS model), inflammation (NO), oxidative stress (ROS), and sebum regulation. Overall, PGC may be regarded as a raw material with “multiefficacy” for cosmetic applications, and the developmental approach employed for PGC may serve as a valuable framework for the advancement of other cosmetic raw materials.

## Author Contributions

Conceptualization: M.Z. and R.L. Design and methodology: W.W. Conduction of the study: W.W., S.Z., Q.Z., Q.Z., Y.S., X.W., and F.H. Statistical analysis and interpretation: W.W. and S.Z. Writing – original draft preparation: W.W. Writing – review and editing: M.Z. and R.L. Resources: M.Z. Supervision: M.Z., R.L., and Y.S. All authors have contributed to and approved the final version of the article submitted for publication.

## Ethics Statement

This study did not involve human or animal subjects, and all cell lines (RAW 264.7, HaCaT, and SZ95) were anonymized commercial cell lines. Furthermore, the human‐derived 3D epidermal models were developed and provided by Guangdong Biocell Biotechnology Co. Ltd. (Guangzhou, China). Their Ethics Committee specifically reviewed and approved the use of donated tissue samples for model construction under protocol number GDLL2024003.

## Conflicts of Interest

All authors, except Ronghua Liu, were employed by Lasur Cosmetics Co. Ltd. The authors declare that the research was conducted without any commercial or financial relationships that could be perceived as potential conflicts of interest. They agree to submit the manuscript to “*Journal of Cosmetic Dermatology*” as an original research article. This manuscript has not been published elsewhere and is not currently under consideration by any other journals.

## Data Availability

The data that support the findings of this study are available from the corresponding author upon reasonable request.

## References

[1] R. J. Hay , N. E. Johns , H. C. Williams , et al., “The Global Burden of Skin Disease in 2010: An Analysis of the Prevalence and Impact of Skin Conditions,” Journal of Investigative Dermatology 134 (2014): 1527–1534. 24166134 10.1038/jid.2013.446

[2] G. Franik , A. Bizoń , S. Włoch , K. Kowalczyk , A. Biernacka‐Bartnik , and P. Madej , “Hormonal and Metabolic Aspects of Acne Vulgaris in Women With Polycystic Ovary Syndrome,” European Review for Medical and Pharmacological Sciences 22 (2018): 4411. 30058676 10.26355/eurrev_201807_15491

[3] V. R. Harris and A. J. Cooper , “Modern Management of Acne,” Medical Journal of Australia 206 (2017): 41–45. 28076744 10.5694/mja16.00516

[4] H. Tsai , W. Lee , P. Wang , K. Cheng , Y. Chen , and S. Shen , “ *Propionibacterium acnes*‐Induced iNOS and COX‐2 Protein Expression via ROS‐Dependent NF‐κB and AP‐1 Activation in Macrophages,” Journal of Dermatological Science 69 (2013): 122–131. 23178030 10.1016/j.jdermsci.2012.10.009

[5] P. Vijayanand , G. Seumois , L. J. Simpson , et al., “Interleukin‐4 Production by Follicular Helper T Cells Requires the Conserved Il4 Enhancer Hypersensitivity Site V,” Immunity 36 (2012): 175–187. 22326582 10.1016/j.immuni.2011.12.014 PMC3288297

[6] K. Kabashima , “New Concept of the Pathogenesis of Atopic Dermatitis: Interplay Among the Barrier, Allergy, and Pruritus as a Trinity,” Journal of Dermatological Science 70 (2013): 3–11. 23473856 10.1016/j.jdermsci.2013.02.001

[7] S. Piazza , G. Martinelli , U. Vrhovsek , et al., “Anti‐Inflammatory and Anti‐Acne Effects of *Hamamelis virginiana* Bark in Human Keratinocytes,” Antioxidants 11 (2022): 1524. 35740016 10.3390/antiox11061119 PMC9220085

[8] J. Hoffmann , F. Gendrisch , C. M. Schempp , and U. Wölfle , “New Herbal Biomedicines for the Topical Treatment of Dermatological Disorders,” Biomedicine 8 (2020): 27. 10.3390/biomedicines8020027 PMC7168306 32046246

[9] A. I. Charles Dorni , A. Amalraj , S. Gopi , K. Varma , and S. N. Anjana , “Novel Cosmeceuticals From Plants—An Industry Guided Review,” Journal of Applied Research on Medicinal and Aromatic Plants 7 (2017): 1–26.

[10] C. A. Espinosa‐Leal and S. Garcia‐Lara , “Current Methods for the Discovery of New Active Ingredients From Natural Products for Cosmeceutical Applications,” Planta Medica 85 (2019): 535–551. 30925621 10.1055/a-0857-6633

[11] Y. Yang , M. Wei , L. Yan , and J. Yang , “Mass Fraction Determination of Total Flavonoids and Total Phenolic Acids in Medicinal and Non‐Medicinal Parts of *Chrysanthemum indicum* L.,” Modified Salt for Chemical Industries 49 (2022): 40–43.

[12] F. He , M. Yu , G. Yao , and J. Zhang , “Determination of Total Phenolic Acids, Flavonoids and Monosaccharide in Chuanshentong Injection,” Journal of Guizhou Medical University 45 (2020): 668–671.

[13] S. Lan , “Content Determination of Total Alkaloids in *Phellodendron amurense* Rupr,” Guangdong Chemical Industry 48 (2021): 165–166.

[14] X. Xu , W. Zhang , C. Huang , et al., “A Novel Chemometric Method for the Prediction of Human Oral Bioavailability,” International Journal of Molecular Sciences 13 (2012): 6964–6982. 22837674 10.3390/ijms13066964 PMC3397506

[15] D. S. Wishart , Y. D. Feunang , A. C. Guo , et al., “DrugBank 5.0: A Major Update to the DrugBank Database for 2018,” Nucleic Acids Research 46 (2018): D1074–D1082. 29126136 10.1093/nar/gkx1037 PMC5753335

[16] M. Shen , S. Tian , Y. Li , et al., “Drug‐Likeness Analysis of Traditional Chinese Medicines: 1. Property Distributions of Drug‐Like Compounds, Non‐Drug‐Like Compounds and Natural Compounds From Traditional Chinese Medicines,” Journal of Cheminformatics 4 (2012): 31. 23181938 10.1186/1758-2946-4-31 PMC3538521

[17] W. Tao , X. Xu , X. Wang , et al., “Network Pharmacology‐Based Prediction of the Active Ingredients and Potential Targets of Chinese Herbal Radix Curcumae Formula for Application to Cardiovascular Disease,” Journal of Ethnopharmacology 145 (2013): 1–10. 23142198 10.1016/j.jep.2012.09.051

[18] D. Otasek , J. H. Morris , J. Bouças , A. R. Pico , and B. Demchak , “Cytoscape Automation: Empowering Workflow‐Based Network Analysis,” Genome Biology 20 (2019): 185. 31477170 10.1186/s13059-019-1758-4 PMC6717989

[19] O. Trott and A. J. Olson , “AutoDock Vina: Improving the Speed and Accuracy of Docking With a New Scoring Function, Efficient Optimization, and Multithreading,” Journal of Computational Chemistry 31 (2010): 455–461. 19499576 10.1002/jcc.21334 PMC3041641

[20] N. Jusoh , J. Ko , and N. L. Jeon , “Microfluidics‐Based Skin Irritation Test Using In Vitro 3D Angiogenesis Platform,” APL Bioengineering 3 (2019): 046106. 10.1063/1.5093975 PMC6697035 31431937

[21] K. Wannita , R. Phetploy , W. Ratjika , et al., “Skin Rejuvenation Efficacy and Safety Evaluation of Kaempferia Parviflora Standardized Extract (BG100) in Human 3D Skin Models and Clinical Trial,” Biomolecules 14 (2024): 776. 39062490 10.3390/biom14070776 PMC11274994

[22] H. Zhou , H. Dai , S. Wang , et al., “Based on In Vitro and 3D Epidermal Skin Model: The Efficacy of *Bletilla striata* Extract for Skin Care,” Journal of Clinical and Cosmetic Dermatology 7 (2024): 203.

[23] Ç. Köksal Karayildirim , “Preparation, Characterization, and Antiangiogenic Evaluation of a Novel 5‐Fluorouracil Derivative Solid Lipid Nanoparticle With a Hen's Egg Chorioallantoic Membrane Assay and Wound Healing Response in HaCaT Keratinocytes,” ACS Omega 9 (2024): 16640–16647. 38617689 10.1021/acsomega.4c00635 PMC11007769

[24] I. M. N. Molagoda , K. T. Lee , Y. H. Choi , and G. Kim , “Anthocyanins From *Hibiscus syriacus* L. Inhibit Oxidative Stress‐Mediated Apoptosis by Activating the Nrf2/HO‐1 Signaling Pathway,” Antioxidants 9 (2020): 42. 31947843 10.3390/antiox9010042 PMC7022859

[25] Y. Lee , Y. Jang , Y. Han , et al., “Aster Glehni Extract Containing Caffeoylquinic Compounds Protects Human Keratinocytes Through the TRPV4‐PPARδ‐AMPK Pathway,” Evidence‐Based Complementary and Alternative Medicine 2018 (2018): 9616574. 30622619 10.1155/2018/9616574 PMC6304624

[26] A. Javed , C. J. Slingerland , T. M. Wood , et al., “Chimeric Peptidomimetic Antibiotic Efficiently Neutralizes Lipopolysaccharides (LPS) and Bacteria‐Induced Activation of RAW Macrophages,” ACS Infectious Diseases 9 (2023): 518–526. 36790385 10.1021/acsinfecdis.2c00518 PMC10012172

[27] D. Torocsik , F. Fazekas , S. Poliska , et al., “Epidermal Growth Factor Modulates Palmitic Acid‐Induced Inflammatory and Lipid Signaling Pathways in SZ95 Sebocytes,” Frontiers in Immunology 12 (2021): 600017. 34025636 10.3389/fimmu.2021.600017 PMC8134683

[28] W. Wang , A. Li , L. Chen , et al., “UPLC‐Q‐TOF‐MS Metabolomic Study on Improvement of Acute Myocardial Ischemia in Rats by *Dalbergia cochinchinensis* Heartwood,” Zhongguo Zhongyao Zazhi 48 (2023): 1043–1053. 36872275 10.19540/j.cnki.cjcmm.20221024.401

[29] C. Vater , A. Apanovic , C. Riethmüller , et al., “Changes in Skin Barrier Function After Repeated Exposition to Phospholipid‐Based Surfactants and Sodium Dodecyl Sulfate In Vivo and Corneocyte Surface Analysis by Atomic Force Microscopy,” Pharmaceutics 13 (2021): 436. 33804924 10.3390/pharmaceutics13040436 PMC8063842

[30] G. Egawa and K. Kabashima , “Barrier Dysfunction in the Skin Allergy,” Allergology International 67 (2018): 3–11. 29153780 10.1016/j.alit.2017.10.002

[31] C. Yang , C. Pan , C. Tseng , and F. Yen , “Antioxidant, Anti‐Inflammation and Antiaging Activities of *Artocarpus altilis* Methanolic Extract on Urban Particulate Matter‐Induced HaCaT Keratinocytes Damage,” Antioxidants 11 (2022): 1514. 36421490 10.3390/antiox11112304 PMC9687219

[32] Y. Kim and K. Lim , “Skin Barrier Dysfunction and Filaggrin,” Archives of Pharmacal Research 44 (2021): 36–48. 33462753 10.1007/s12272-021-01305-x

[33] J. Sharifi‐Rad , S. M. Hoseini‐Alfatemi , M. Sharifi‐Rad , and J. A. Teixeira da Silva , “Antibacterial, Antioxidant, Antifungal and Anti‐Inflammatory Activities of Crude Extract From Nitraria Schoberi Fruits,” 3 Biotech 5 (2015): 677–684. 10.1007/s13205-014-0266-1 PMC4569636 28324518

[34] A. Balato , S. Cacciapuoti , R. Di Caprio , et al., “Human Microbiome: Composition and Role in Inflammatory Skin Diseases,” Archivum Immunologiae et Therapiae Experimentalis (Warsz) 67 (2019): 1–18. 10.1007/s00005-018-0528-4 30302512

[35] S. L. Prescott , D. Larcombe , A. C. Logan , et al., “The Skin Microbiome: Impact of Modern Environments on Skin Ecology, Barrier Integrity, and Systemic Immune Programming,” World Allergy Organization Journal 10 (2017): 29. 28855974 10.1186/s40413-017-0160-5 PMC5568566

[36] T. Yuki , H. Yoshida , Y. Akazawa , A. Komiya , Y. Sugiyama , and S. Inoue , “Activation of TLR2 Enhances Tight Junction Barrier in Epidermal Keratinocytes,” Journal of Immunology 187 (2011): 3230–3237. 10.4049/jimmunol.1100058 21841130

[37] S. Naik , N. Bouladoux , C. Wilhelm , et al., “Compartmentalized Control of Skin Immunity by Resident Commensals,” Science 337 (2012): 1115–1119. 22837383 10.1126/science.1225152 PMC3513834

[38] Y. C. Giam , A. A. Hebert , M. V. Dizon , et al., “A Review on the Role of Moisturizers for Atopic Dermatitis,” Asia Pacific Allergy 6 (2016): 120–128. 27141486 10.5415/apallergy.2016.6.2.120 PMC4850335

[39] N. F. Agamia , K. F. El Mulla , N. M. Alsayed , et al., “Isotretinoin Treatment Upregulates the Expression of p53 in the Skin and Sebaceous Glands of Patients With Acne Vulgaris,” Archives of Dermatological Research 315 (2023): 1355–1365. 36585988 10.1007/s00403-022-02508-y PMC10205870

[40] V. Shtilbans , M. Wu , and D. E. Burstein , “Current Overview of the Role of Akt in Cancer Studies via Applied Immunohistochemistry,” Annals of Diagnostic Pathology 12 (2008): 153–160. 18325479 10.1016/j.anndiagpath.2007.12.001

[41] C. Couture , P. Desjardins , K. Zaniolo , L. Germain , and S. L. Guérin , “Enhanced Wound Healing of Tissue‐Engineered Human Corneas Through Altered Phosphorylation of the CREB and AKT Signal Transduction Pathways,” Acta Biomaterialia 73 (2018): 312–325. 29656072 10.1016/j.actbio.2018.04.021

[42] T. Cong , D. Hao , X. Wen , X. Li , G. He , and X. Jiang , “From Pathogenesis of Acne Vulgaris to Anti‐Acne Agents,” Archives of Dermatological Research 311 (2019): 337–349. 30859308 10.1007/s00403-019-01908-x

[43] M. H. Koo , H. Chae , J. H. Lee , S. Suh , and U. J. Youn , “Anti‐Inflammatory Lanostane Triterpenoids From *Ganoderma lucidum* ,” Natural Product Research 35 (2021): 4295–4302. 31872776 10.1080/14786419.2019.1705815

[44] S. Yoon , J. Park , S. Choi , et al., “Ginsenoside Rg3 Regulates S‐Nitrosylation of the NLRP3 Inflammasome via Suppression of iNOS,” Biochemical and Biophysical Research Communications 463 (2015): 1184–1189. 26086107 10.1016/j.bbrc.2015.06.080

[45] J. Zhou , J. Zhang , J. Li , et al., “Ginsenoside F2 Suppresses Adipogenesis in 3T3‐L1 Cells and Obesity in Mice via the AMPK Pathway,” Journal of Agricultural and Food Chemistry 69 (2021): 9299–9312. 34342980 10.1021/acs.jafc.1c03420

[46] Y. Jeong and Y. Park , “Ergosterol Peroxide From the Medicinal Mushroom *Ganoderma lucidum* Inhibits Differentiation and Lipid Accumulation of 3T3‐L1 Adipocytes,” International Journal of Molecular Sciences 21 (2020): 460. 31936890 10.3390/ijms21020460 PMC7014426

[47] K. Shin , S. J. Choe , Y. Uchida , I. Kim , Y. Jeong , and K. Park , “Ginsenoside Rb1 Enhances Keratinocyte Migration by a Sphingosine‐1‐Phosphate‐Dependent Mechanism,” Journal of Medicinal Food 21 (2018): 1129–1136. 30148701 10.1089/jmf.2018.4246 PMC6913107

[48] W. Xia , Z. Zhu , S. Xiang , and Y. Yang , “Ginsenoside Rg5 Promotes Wound Healing in Diabetes by Reducing the Negative Regulation of SLC7A11 on the Efferocytosis of Dendritic Cells,” Journal of Ginseng Research 47 (2023): 784–794. 38107390 10.1016/j.jgr.2023.06.006 PMC10721477

[49] Z. Li , R. Jiang , M. Wang , et al., “Ginsenosides Repair UVB‐Induced Skin Barrier Damage in BALB/c Hairless Mice and HaCaT Keratinocytes,” Journal of Ginseng Research 46 (2022): 115–125. 35035244 10.1016/j.jgr.2021.05.001 PMC8753432

[50] C. F. Carson , K. A. Hammer , and T. V. Riley , “ *Melaleuca alternifolia* (Tea Tree) Oil: A Review of Antimicrobial and Other Medicinal Properties,” Clinical Microbiology Reviews 19 (2006): 50–62. 16418522 10.1128/CMR.19.1.50-62.2006 PMC1360273

[51] W. Wang , M. Luo , Y. Fu , S. Wang , T. Efferth , and Y. Zu , “Glycyrrhizic Acid Nanoparticles Inhibit LPS‐Induced Inflammatory Mediators in 264.7 Mouse Macrophages Compared With Unprocessed Glycyrrhizic Acid,” International Journal of Nanomedicine 8 (2013): 1377–1383. 23610519 10.2147/IJN.S37788 PMC3629880

[52] Z. Yang , Y. Zhang , E. Lazic Mosler , et al., “Topical Benzoyl Peroxide for Acne,” Cochrane Database of Systematic Reviews 3 (2020): CD011154. 32175593 10.1002/14651858.CD011154.pub2 PMC7077870

[53] K. Lintner , C. Mas‐Chamberlin , P. Mondon , O. Peschard , and L. Lamy , “Cosmeceuticals and Active Ingredients,” Clinics in Dermatology 27 (2009): 461–468. 19695477 10.1016/j.clindermatol.2009.05.009

[54] A. H. Mota , P. Rijo , J. Molpeceres , and C. P. Reis , “Broad Overview of Engineering of Functional Nanosystems for Skin Delivery,” International Journal of Pharmaceutics 532 (2017): 710–728. 28764984 10.1016/j.ijpharm.2017.07.078

